# Mitochondrial Transfer Rescues Respiration to Support *De Novo* Pyrimidine Biosynthesis and Tumor Progression

**DOI:** 10.1158/0008-5472.CAN-24-0737

**Published:** 2025-11-17

**Authors:** Maria Dubisova, Klara Bohacova, Zuzana Nahacka, Daniel Kraus, Jaromir Novak, Sarka Dvorakova, Petra Brisudova, Natalie Danesova, Saba Selvi, Mariia Hrysiuk, Berwini B. Endaya, Panagiotis Botsios, Dan-Diem Thi Le, Monika Novotna, Sona Vodenkova, Jaroslav Truksa, Karel Chalupsky, Krystof Klima, Jan Prochazka, Radislav Sedlacek, Francesco Mengarelli, Patrick Orlando, Luca Tiano, Stepana Boukalova, Michael V. Berridge, Renata Zobalova, Jiri Neuzil

**Affiliations:** 1 Institute of Biotechnology, Czech Academy of Sciences, Prague-West, Czech Republic.; 2 Faculty of Science, Charles University Prague, Czech Republic.; 3 Institute of Experimental Medicine, Czech Academy of Sciences, Prague, Czech Republic.; 4 Biomedical Centre, Faculty of Medicine in Pilsen, Charles University, Pilsen, Czech Republic.; 5 First Faculty of Medicine, Charles University, Prague, Czech Republic.; 6 Institute of Molecular Genetics, Czech Academy of Sciences, Prague, Czech Republic.; 7 Department of Life and Environmental Sciences, Polytechnic University of Marche, Ancona, Italy.; 8 Malaghan Institute of Medical Research, Wellington, New Zealand.; 9 School of Pharmacy and Medical Science, Griffith University, Southport, Australia.

## Abstract

Cancer cells with severe defects in mitochondrial DNA (mtDNA) can import mitochondria via horizontal mitochondrial transfer to restore respiration. Mitochondrial respiration is necessary for the activity of dihydroorotate dehydrogenase (DHODH), an enzyme of the inner mitochondrial membrane that catalyzes the fourth step of *de novo* pyrimidine synthesis. In this study, we investigated the role of *de novo* synthesis of pyrimidines in driving tumor growth in mtDNA-deficient (ρ^0^) cells. Although ρ^0^ cells grafted in mice readily acquired mtDNA, this process was delayed in cells transfected with alternative oxidase (AOX), which combines the functions of mitochondrial respiratory complexes III and IV. The ρ^0^ AOX cells were glycolytic but maintained normal DHODH activity and pyrimidine production. Deletion of DHODH in a panel of tumor cells completely blocked or delayed tumor growth. The grafted ρ^0^ cells rapidly recruited tumor-promoting/stabilizing cells of the innate immune system, including protumor M2 macrophages, neutrophils, eosinophils, and mesenchymal stromal cells (MSC). The ρ^0^ cells recruited MSCs early after grafting, which were potential mitochondrial donors. Grafting MSCs together with ρ^0^ cancer cells into mice resulted in mitochondrial transfer from MSCs to cancer cells. Overall, these findings indicate that cancer cells with compromised mitochondrial function readily acquire mtDNA from other cells in the tumor microenvironment to restore DHODH-dependent respiration and *de novo* pyrimidine synthesis. The inhibition of tumor growth induced by blocking DHODH supports targeting pyrimidine synthesis as a potential widely applicable therapeutic approach.

**Significance:**

Mitochondrial complexes III and IV promote tumor progression by supporting *de novo* pyrimidine synthesis, requiring cancer cells devoid of mitochondrial DNA to recruit mitochondria from source cells to restore respiration in order to form tumors.

## Introduction

Cancer is the second most common reason for premature death, with its incidence still on the rise, in particular due to serious therapeutic limitations (1). There are a number of reasons why tumor cells escape treatment, such as high-level metabolic plasticity (2), the existence of cancer stem cells (also referred to as tumor-initiating cells; ref. 3), and the recently discovered phenomenon of horizontal mitochondrial transfer (HMT; ref. 4). The process of HMT between mammalian cells was initially documented in an *in vitro* system (5) and later shown for mouse models of cancer (6), with reports pointing to its relevance in cancer therapy (7, 8). In addition to experimental cancer settings, HMT has also been shown for canine transmissible venereal tumors, in which it occurs over long periods of time (9–11). Due to its importance in malignant pathologies, HMT could be considered a new hallmark of cancer (12).

HMT, a process that involves “donor” and “acceptor” cells, provides intercellular, mitochondria-mediated communication by means of multiple mechanisms (4), including tunneling nanotubes (TNT; refs. 5, 13), extracellular vesicles (14, 15), and gap junctions (16, 17). Functionally, cancer cells with defects in mitochondrial DNA (mtDNA) invoke HMT from stromal cells in order to restore respiration. This was shown initially in coculture of mtDNA-deficient (ρ^0^) lung cancer cells and mesenchymal stem cells (MSC) or fibroblasts, which resulted in the renewal of functional mitochondrial respiration (18). To date, there have been a number of studies demonstrating that MSCs serve as a major donor of mitochondria under both physiologic and pathologic conditions (4). In our previous study, we observed that grafting murine ρ^0^ tumor cells into mice resulted in the generation of syngeneic tumors only after acquisition of mtDNA (6) by way of transfer of whole mitochondria from stromal cells (19).

We then queried the link between renewal of respiration and tumor growth. Of note, ρ^0^ cells that do not respire make enough ATP by glycolysis to support rapid cell growth (20), which is consistent with the notion that the formation of ATP by glycolysis is kinetically superior to its formation by oxidative phosphorylation (OXPHOS; ref. 21). Mitochondrial respiration, therefore, does not seem to be critical for ATP generation under these conditions. Further research showed that mitochondrial respiration, a major component of OXPHOS, is necessary for the activity of dihydroorotate dehydrogenase (DHODH), an enzyme of the inner mitochondrial membrane that catalyzes the fourth step of *de novo* pyrimidine synthesis (20, 22). This is supported by research showing the importance of *de novo* pyrimidine synthesis in tumor growth, especially during its early stages (23). Furthermore, the absence of DHODH causes S-phase arrest early in the cell cycle, stalling cell proliferation (20, 23). This points to DHODH being a potential target for cancer therapy (24–26). Linked to this, it has been shown that during malignant conversion, expression of DHODH increases during early stages, i.e., during hyperplasia (27), pointing to its importance in tumor growth.

In this study, we investigated whether DHODH is a prerequisite for tumor growth in a breast cancer cell model with and without mtDNA. In the absence of mtDNA, these cells retain full tumor-forming potential following mitochondrial acquisition. For this project, we used the 4T1 ρ^0^ breast cancer cell line and its counterparts, stably transfected with alternative oxidase (AOX), and found that HMT was delayed in cells expressing this enzyme that has functions of both complex III (CIII) and complex IV (CIV; ref. 27). We observed that deficiency in DHODH considerably delays or blocks tumor growth. We also observed that early migration of immune cells and MSCs into ρ^0^ tumor cell stroma likely supports tumor growth, with MSCs serving as donors of functional mitochondria. Our data show that respiration, more specifically its components CIII and CIV, play a major role in the onset of efficient tumor growth, reinforcing the notion that both DHODH and CIII/CIV inhibitors may function as efficient, broad-spectrum anticancer drugs. Moreover, inhibiting HMT to tumor cells with impaired mitochondrial respiration could increase the effectiveness of anticancer treatment directed at mitochondria.

## Materials and Methods

### Cell culture

4T1 mouse triple-negative metastatic breast cancer cells were purchased from the ATCC. The cells were cultivated in the RPMI 1640 medium with 10% FBS and antibiotics, 1 mmol/L pyruvate, 50 μg/mL uridine, and 5 mg/mL glucose. 4T1 ρ^0^, 4T1 ρ^0^ AOX, and 4T1 DHODH knockout (KO) cells were prepared as described (6, 20). The sublines derived from 4T1, 4T1 ρ^0^, and 4T1 ρ^0^ AOX cells were prepared as described (20). Briefly, 4T1, 4T1 ρ^0^, and 4T1 ρ^0^ AOX cells were injected subcutaneously into Balb/c mice at 10^6^ cells per animal. On the required day, mice were sacrificed, tumors were excised, and cells were isolated from the tissue in the presence of 6TG as described (6). The isolated cells were used to establish sublines for further study. The generated cell lines were stable in culture over extended periods of time, maintaining their proliferation and mtDNA status (Supplementary Fig. S1D and S1F). Human colorectal cancer HCT116, triple-negative breast cancer MDA-MB-231, and pancreatic cancer PaTu 8902 cells were purchased from the ATCC. HCT116, MDA-MB-231, and PaTu 8902 DHODH^KO^ cells were prepared as described in Materials and Methods. Cells were cultivated in DMEM with 10% FBS and antibiotics, 1 mmol/L pyruvate, 50 μg/mL uridine, and 5 mg/mL glucose. All cell lines used were *Mycoplasma*-free, as verified by their periodical testing using the MycoAlert Mycoplasma Detection Kit (Lonza Bioscience). Cells were tested a week after defrosting and then every 3 weeks. The last date the cells were tested is September 9, 2025. Cells were used for up to 25 passages, at which stage a fresh batch of cells was put into culture.

### Preparation of 4T1 GFP and 4T1 ρ^0^ GFP cells and lentivirus production

4T1 GFP and 4T1 ρ^0^ GFP cells with stable expression of cytoplasmic GFP were obtained by transduction with lentiviral particles bearing GFP plasmid (Addgene). For preparation of recombinant lentiviruses, calcium–phosphate transfection of HEK293T cells with psPAX2 (Addgene, #12260) and pMD2.G together (Addgene, #12259) with the pKAM-GFP plasmid (Addgene, #101865) was used. The medium containing the lentiviral particles was harvested 48 hours after transfection, and the viral particles were precipitated using the virus precipitation solution PEG-it (System Biosciences). 4T1 and 4T1 ρ^0^ cells were transduced with viruses at a multiplicity of infection of 5 to 10. Finally, the transduced cells were selected by sorting for GFP.

### Preparation of cells with DHODH^KO^

Genomic deletion of *DHODH* gene in MDA-MB-231, HCT116, and PaTu 8902 cells was performed using a CRISPR-Cpf1/Cas12 system (28). Identification of crRNAs targeting exon 2 was determined in the Ensembl database and the CRISPOR software. These were designed as 3 crRNAs (5′→3′ CCC ACA CAA ATG TCA GAA TGT TC, TAT GCT GAA CAC CTG ATG CCG AC, and AAA AAG ATC AGT CCC AGC TGA GA) interspaced with the protospacer adjecent motif (PAM) sequence AAT TTC TAC TCT TGT AGA T specific for Cas12. DNA oligonucleotides were annealed (37°C/30 minutes, 95°C/5 minutes, and 25°C ramp down at 5%/minute) and ligated using T4 DNA ligase to the pX AsCpf1-Venus-NLS crRNA plasmid previously digested with FastDigest BpiI enzyme. All enzymes were purchased from Thermo Fisher Scientific, and enzymatic reactions were proceeded according to the manufacturer’s protocol. DNA cloning was followed by transformation of the Endura bacterial strain (VWR).

Transformation was achieved by incubation for 30 minutes on ice and exposure to heat-shock for 1 minute at 42°C, followed by placing the samples on ice for 2 minutes. Bacteria were used for inoculation of LB media, and they were cultivated at 37°C at 200 rpm. Transformed bacteria were plated on ampicillin agarose plates. Positive colonies were picked by colony PCR using the DreamTaq Green PCR Master Mix (2X) and Cpf1-specific primers (forward pXPR: GGA CTA TCA TAT GCT TAC CGT AAC TTG AAA G; reverse sgRNA ivt R: AAA AGC ACC GAC TCG GTG CC). The PCR reaction was conducted according to the manufacturer’s protocol, and products of end-point PCR were verified by DNA electrophoresis on an agarose gel (2% agarose, 60 V/90 minutes).

Positive clones were used for inoculating 100 mL of LB media and cultivated overnight at 200 rpm at 37°C. Plasmid DNA was isolated using the NucleoBond Xtra Midi Kit (Macherey-Nagel) according to the manufacturer’s protocol. The Cpf1 vector with crRNA targeted to exon 2 of the *DHODH* gene was verified by Sanger sequencing (Eurofins Genomics; forward pXPR: GGA CTA TCA TAT GCT TAC CGT AAC TTG AAA G or reverse sgRNA ivt R: AAA AGC ACC GAC TCG GTG CC). The verified vector was then used for Lipofectamine transfection of MDA-MB-231, HCT116, and PaTu 8902 cell lines.

Transfection of cells was performed using Lipofectamine 3000 (Thermo Fisher Scientific) according to the manufacturer’s protocol, with 2 μg of plasmid DNA used for the transfection reaction. After 48 hours, transfected cells were subjected for single-cell sorting for YFP (Venus Remedies) positivity and then analyzed using PCR and DNA electrophoresis. To verify the KO status of the *DHODH* gene on the level of genomic DNA, we used one forward primer (CAT GCC TGT AAT CCC AGC ACT TTG G) and two reverse primers targeting the exon 3 sequence after the PAM3 sequence (reverse 1: TGG TCC TGC TAC CTC CTA GC) or between PAM2 and PAM3 sequences (reverse 2: GAC TGC AGC CTT CGC CTT TGT A). Positive clones were confirmed by Western blotting and Sanger sequencing. For sequencing, cells were seeded on six-well plates, and DNA was isolated using the Wizard SV Genomic DNA Purification System, followed by polymerization by means of the DreamTaq PCR Master Mix (2X) and purification from the agarose gel using a Gel and PCR Clean-up kit (Macherey-Nagel). PCR and sequencing reactions were performed the same way as described above using the forward (GTA CAG TAC ACA TTT GGA GGC AAG A) or reverse primer (TCT CCT CAG CTC TCA AAC TGA CC).

### Evaluation of uridine auxotrophy

Cells were plated in six-well plates at 5 × 10^3^ per well and cultured in RPMI 1640 medium containing either 10% FBS and antibiotics alone or supplemented with 1 mmol/L pyruvate, 50 μg/mL uridine, and 5 mg/mL glucose for 72 hours at 37°C in 5% CO_2_ atmosphere. Duplicate cell samples were rinsed with PBS and harvested using trypsinization, and the cell number was quantified using a CASY Cell Counter (Roche).

### mtDNA level assay

DNA was extracted using the E.Z.N.A. Tissue DNA kit following the manufacturer’s instructions. DNA was quantified with the NanoDrop 8000 UV/Vis Spectrophotometer (Thermo Fisher Scientific) and combined with forward and reverse primers (nDNA, mB2M forward 5′-ATG GGA AGC CGA ACA TAC TG-3′, reverse 5′-CAG TCT CAG TGG GGG TGA AT-3′ and mtDNA; mMito1 forward 5′-CTA GAA ACC CCG AAA CCA AA-3′, reverse 5′-CCA GCT ATC ACC AAG CTC GT-3′) and PerfeCTa SYBR Green SuperMix (Quantabio). The reaction was run on the Eco qPCR System (Illumina) with the following settings: one cycle of 50°C for 2 minutes, one cycle of 95°C for 10 minutes, and 40 cycles of 95°C for 15 seconds and 60°C for 1 minute.

### mtDNA sequencing

Total DNA containing mtDNA was isolated via the DNAzol reagent (Molecular Research Center) according to the manufacturer’s instructions. Full-length mtDNA was sequenced by amplifying 10 1.8 kb dsDNA PCR products using the 5× HOT FIREPol Blend Master Mix with 10 mmol/L MgCl_2_. PCR products were then separated on agarose gels to confirm the specificity of the reaction and were purified using the DNA Clean & Concentrator Kit (Zymo Research). Purified PCR products (125 ng) were combined with water and 1 µL of 25 µmol/L primer stocks to yield 10 µL sequencing reaction mixture. Sanger sequencing was performed via Eurofins Genomics Light Run service. Sequences were aligned using the SeqMan Pro suite from the DNASTAR Lasergene 17 software enabling the visualization of the peak histograms.

### RNA extraction

Total RNA was extracted using the Total RNA Purification Kit (Norgen Biotek, #17200) according to the manufacturer’s protocol. Total RNA was diluted in 20 μL of elution buffer (included in the kit). RNA Clean & Concentrator-5 (Zymo Research, #R1013) was used to remove contaminating genomic DNA. DNase I treatment was done following the manufacturer’s instructions (Appendix - In-column DNase I treatment). The final total RNA was diluted in 20 μL of DNase/RNase-free water (included in the kit). The concentration of total RNA was assessed using a spectrophotometer (NanoDrop 2000, Thermo Fisher Scientific), and the quality of the RNA was assessed using Fragment Analyzer 5200 (Agilent, RNA 15 nt Kit, #DNF-471). The RNA quality number was above 8 for all samples.

### Library preparation

Total RNA (100 ng) was used for the library preparation using the QuantSeq 3′ mRNA-Seq V2 Library Prep Kit with UDI (Lexogen, #192.24) according to the manufacturer’s protocol. Library qualities were assessed using the Fragment Analyzer 5200 (Agilent, High Sensitivity NGS Fragment Kit, #DNF-474), and concentrations were determined by the Qubit 4 Fluorometer (Thermo Fisher Scientific, dsDNA Quantification Assay Kit, #Q32854). Libraries were sequenced using NextSeq500 Mid Output 150 cycles (Illumina) with 144 cycles.

### Transcriptomics analysis

Reads were filtered for low-quality reads and adapter sequences using Cutadapt (v. 3.5). The cleaned reads were then aligned using HISAT2 (v. 2.2.1) to the mouse genome version GRCm39 and annotation version GCF_000001635.27. A count table was then generated using FeatureCounts (v. 2.0.3). The counts were normalized and analyzed using pyDESeq2 (v. 0.3.5) in Python (v. 3.11). Differential gene expression was assessed across all conditions for each experiment.

### Evaluation of respiration

Routine and DHODH-dependent respiration was assessed using the high-resolution Oxygraph-2k respirometer (Oroboros Technologies), as previously published (20).

### Isolation of mitochondria

To isolate mitochondria, cells were suspended in the isolation buffer (250 mmol/L sucrose, 10 mmol/L Tris, and 1 mmol/L EDTA) and passed 3 times through an 8-µm tungsten carbide ball of a Balch homogenizer (Isobiotec cell homogenizer) using a hand-driven 1-mL syringe. The homogenate was centrifuged at 800 *g* for 5 minutes at 4°C, and the supernatant was collected and precleared by centrifugation at 3,000 *g* for 5 minutes followed by centrifugation at 10,000 *g* for 15 minutes. Pelleted mitochondria were washed once with the isolation buffer and stored at −80°C.

### DHODH activity

Activity of DHODH was assessed using a modified protocol derived from a published method (29). Briefly, isolated mitochondria were resuspended in hypotonic buffer (10 mmol/L Tris-HCl, pH 7.6) and lysed using three freeze–thaw cycles. Lysates were incubated in a solution of 184 mmol/L K_2_CO_3_.HCl (pH 8.0), 500 µmol/L DHO, and 80 µmol/L coenzyme Q_1_ for 60 minutes at 37°C, and the reference sample was kept on ice. The reaction mixture was subsequently supplemented with 20 mmol/L K_2_CO_3_, 2 mmol/L K_3_[Fe(CN)_6_], and 1 mmol/L 4-(trifluoromethoxy)benzamidoxime and heated for 4 minutes at 80°C. Reactions were terminated by rapid cooling on ice, and fluorescence intensity was read using the Infinite M200 plate reader (Tecan) at excitation and emission wavelengths of 320 and 420 nm, respectively.

### NADH/NAD^+^ ratio

The levels of oxidized and reduced nicotinamide adenine dinucleotides (NAD^+^ and NADH) were measured in cells plated at a density of 1.5 × 10^4^ per well in white-walled 96-well plates. The bioluminescent NADH/NAD^+^ Glo Assay (Promega) was used for the analysis, and luminescence was detected using a Tecan Infinite M200 Microplate Reader.

### Western blotting 

Cells were lysed in the RIPA buffer supplemented with the Complete Protease Inhibitor Cocktail (SERVA Electrophoresis). Lysates were clarified by centrifugation, and total proteins were determined using the bicinchoninic acid (BCA) method. Total protein (35–50 mg) of was resolved by SDS-PAGE and transferred to nitrocellulose membranes, which were then probed with the following antibodies: anti–β-actin (8H10D10) from Cell Signaling Technology, anti-DHODH IgGfrom Santa Cruz Biotechnology (sc-166348), and custom-made anti-AOX IgG from 21st Century Biochemicals.

### Native blue gel electrophoresis

Ten μg of digitonin-solubilized (8 g/g protein) mitochondria were mixed with the sample loading buffer (0.015 μL per 1 μg protein, 0.75 mol/L aminocaproic acid, 50 mmol/L Bis-Tris, 12% glycerol, 0.5 mmol/L EDTA, and 5% Coomassie brilliant blue G-250) and separated on 3% to 12% and 4% to 16% NativePAGE Novex Bis-Tris gradient gels (Invitrogen). Electrophoresis ran in three steps, i.e., using the blue cathode buffer (0.02% Coomassie brilliant blue G-250) at 35 V for 70 minutes and then the clear cathode buffer at 25 V overnight. Finally, the voltage was increased to 200 V for 2 hours. For Western blotting, gels were incubated in the transfer buffer for 10 minutes with 0.1% SDS, and proteins were transferred to polyvinylidene difluoride 0.2-μm membranes (Bio-Rad). Subsequent Western blotting analysis used the same procedure as was performed as for SDS-PAGE using the following antibodies: complex I (CI) – anti-NDUFA9 (Abcam, ab14713), complex II (CII) – anti-SDHA IgG (Abcam, ab14715), anti-SDHB IgG (Abcam, 14714), and complex V (CV) – anti-ATP5B IgG (Sigma-Aldrich, HPA001250); anti-Hsp60 IgG (Cell Signaling Technology, 12165) was used as a loading control.

### Flow cytometry

Tumors were digested with enzymes from the Mouse Tumor Dissociation Kit (130-096-730, Miltenyi Biotec) for 10 minutes at 37°C and minced in a gentleMACS Dissociator (Miltenyi Biotec) according to the manufacturer’s instructions. Cells were washed with the Flow cytometry (FCM) buffer (2% FCS and 10 mmol/L EDTA in HBSS without Ca^2+^ and Mg^2+^) and filtered using a 100-μm cell strainer. Red blood cells were lysed using the ACK buffer. Up to 2.5 × 10^6^ cells were taken for the FCM analysis. To exclude dead cells from subsequent analysis, cell suspensions were first stained with Fixable Viability Dye eFluor455UV (Thermo Fisher Scientific). Next, surface antigens were labeled (30 minutes on ice) with antibodies listed in Supplementary Table S1 diluted in the FCM buffer supplemented with 20% BD Horizon Brilliant Stain Buffer (BD Biosciences) and an Fc-receptor blocking antibody (2.4G2). Surface-labeled cells were then fixed using eBioscience Fixation/Permeabilization buffers (00-5523-00, Thermo Fisher Scientific) and were stained with intracellular antigens (CD206 and FoxP3) for 30 minutes at room temperature. Breast cancer cells were detected using anti-CD24 IgG, and anti-CD49f FCM data were acquired on Cytek Aurora flow cytometer (5L 16UV-16V-14B-10YG-8R, Cytek Biosciences).

### Confocal microscopy

For microscopic detection of CD90 and Sca1 markers in tumor tissue, whole resected tumors were fixed in 3.7% paraformaldehyde for 12 to 24 hours (based on tissue size), soaked with 30% sucrose (Serva Electrophoresis) solution, snap-frozen in Tissue-Tek O.C.T. Compound (Sakura Finetek), and stored at −80°C. Cryosections (5 μm thick) were cut using a microtome (Leica Biosystems), subsequently labeled with anti-CD90 and anti–Sca1 or anti-CD45 antibodies and corresponding fluorescent secondary antibodies, and mounted in Mowiol containing Hoechst 33342 (Sigma-Aldrich). Antibodies used were anti-CD90 IgG (PA5-80127, Invitrogen), anti–Sca1 IgG (ab51317, Abcam), goat anti-rat secondary IgG, Alexa Fluor Plus 488 (A48262), and goat anti-rabbit secondary IgG, Alexa Fluor Plus 647 (A32733, both Invitrogen).

Samples were imaged on a Nikon ECLIPSE Ti2 microscope with Yokogawa CSU-W1 confocal unit (Nikon), and fluorescence was excited with 405-, 488-, and 633-nm lasers (emission filters 455/50, 525/50 and 690/50), with exposure times 200, 100, and 200 ms, respectively. From each section, at least two fields of view (size 575 × 575 × 10 μm with z-spacing of 0.5 μm) were captured, and maximum intensity projection images were further visualized using Fiji software.

### Isolation of mito::mKate2 mesenchymal stromal cells

Mesenchymal stromal cells (MSC) were prepared from the bone marrow of Balb/c mito::mKate2 mice bearing heterozygous, mitochondria-specific expression of the far-red fluorescent protein mKate2. The isolation process used the MesenCult Expansion Kit (Scintilla Pharmaceuticals) based on the manufacturer’s protocol. In summary, femurs and tibias were removed from euthanized mice, the bone marrow was flushed and resuspended in MesenCult expansion media with MesenPure, and the suspension was then seeded in cell culture flasks. The cells were subsequently cultured for up to 10 passages. MSCs obtained from passages 3 to 10 were used in experiments.

### Formation of tumors

4T1, 4T1 ρ^0^, and 4T1 ρ^0^ AOX cells were injected subcutaneously into the right flank of Balb/c mice at 10^6^ per animal. HCT116, MDA-MB-231, and PaTu 8902 cells and their DHODH^KO^ counterparts were injected at 10^6^ per animal into the right flank of NOD/SCID gamma (NSG) mice. Tumor growth was monitored twice a week using calipers. The experiment was terminated once tumors reached the size of 5% of the animal’s volume. Animal studies were approved by and performed according to the guidelines of the Czech Animal Ethics Committee.

### *In vivo* coculture

To monitor mitochondrial transfer in an *in vivo* coculture system, Balb/c mice were grafted subcutaneously with a mixture of MSC mito::mKate2 containing heterozygous expression of far-red fluorescent protein mKate2 and 4T1 GFP or 4T1 ρ^0^ GFP cells at the ratio of 1:3 in 100 μL of Geltrex (Thermo Fisher Scientific, A1413301). MSCs and 4T1 cells lacking the fluorescent protein were used as controls for fluorescence background. For microscopy analysis, tumors were harvested on different days after tumor cell injection (days 5 and 15), tumors were dissected and seeded into glass-bottom Petri dishes (Cellvis). After indicated time points, attached cells that migrated from the tumor tissue were imaged using the Nikon ECLIPSE Ti2 microscope with the Yokogawa CSU-W1 confocal unit (Nikon). Excitation was achieved with 488- and 594-nm lasers (emission filters 525/50 and 645/75), and double-positive cells were imaged at ×25 or ×100 magnification (pixel size 0.28 or 0.07 μm). Images were processed (maximum intensity projection and contrast adjustment) in FIiji software.

### Statistical analysis

Unless specified otherwise, the data are presented as mean values ± SD derived from a minimum of three independent experiments. For mouse experiments, groups comprised six animals unless otherwise noted. Statistical significance was assessed using the two-tailed unpaired Student *t* test, with *P* < 0.05 considered significant, using GraphPad Prism software. ANOVA was utilized for multiple comparisons when applicable. Images shown are representative of results obtained from at least three independent experiments. Statistical signification is as follows: *, *P* value of 0.01 to 0.05; **, *P* value of 0.001 to 0.01; ***, *P* value of 0.0001 to 0.001; ****, *P* < 0.0001. Statistically significant differences were achieved when *P* value < 0.05.

### Evaluation of ATP level

The level of ATP was assessed using the CellTiter-Glo Luminescent Assay (Promega) according to the manufacturer’s instructions. Cells were seeded in 96-well plates in the absence or presence of 50 mmol/L 2DG, and the ATP level was assessed and normalized to the total protein level in cell lysates.

### qRT-PCR

Total RNA was extracted from 4T1, 4T1 ρ^0^, and 4T1 ρ^0^ AOX cells in triplicates using RNAzol (Molecular Research Center), and its purity and concentration were assessed using NanoDrop (Thermo Fisher Scientific). One microgram of RNA was transcribed to cDNA using the RevertAid RT Reverse Transcription Kit (Thermo Fisher Scientific) with random hexamer primer. To assess the expression of target selected genes, using published primer sequences (19), 250 ng of cDNA was used for each qRT-PCR reaction containing PerfeCTa SYBR Green SuperMix (Quantabio). The reaction was run on the Eco qPCR system (Illumina) with the following setting: initial denaturation (95°C for 12 minutes) followed by 38 cycles of denaturation, annealing, and extension (95°C for 15 seconds, 60°C for 20 seconds, and 72°C for 20 seconds, respectively). Target genes were normalized to the housekeeping gene (actin), and changes in gene expression were determined using the ΔΔCt method.

### Proteomics

#### Protein digestion

Cell pellets were lysed by boiling at 95°C for 10 minutes in 100 mmol/L triethylammonium bicarbonate (TEAB) containing 2% sodium deoxycholate (SDC), 40 mmol/L chloroacetamide, and 10 mmol/L tris(2-carboxyethyl)phosphine and further sonicated (BANDELIN Sonopuls Mini 20, MS 1.5). Protein concentration was determined using the BCA protein assay kit, and 30 µg of protein per sample was used for mass spectrometry (MS) sample preparation. The sample volume was then adjusted to 50 µL in total by adding 100 mmol/L TEAB containing 2% SDC.

Samples were further processed using SP3 beads as published (30). Briefly, 5 µL of SP3 beads was added to 30 µg of protein in the lysis buffer and filled to 50 µL with 100 mmol/L TEAB. Protein binding was induced by the addition of ethanol to 6% (v/v) final concentration. Samples were mixed and incubated for 5 minutes at room temperature. The tubes were then placed into a magnetic rack, and the supernatant was discarded. Beads were subsequently washed two times with 180 µL of 80% ethanol. After washing, samples were digested with trypsin (trypsin/protein ration 1/30) and reconstituted in 100 mmol/L TEAB at 37°C overnight. After digestion, samples were acidified with trifluoroacetic acid (TFA) to 1% final concentration, and peptides were desalted using in-house made stage tips packed with C18 disks (Empore; ref. 31).

#### Nanoscale LC-MS 2 analysis

Nano reversed phase columns (EASY-Spray column, 50 cm × 75 µm ID, PepMap C18, 2 µm particles, 100 Å pore size) were used for LC/MS analysis. Mobile phase buffer A was composed of water and 0.1% formic acid. Mobile phase B was composed of acetonitrile and 0.1% formic acid. Samples were loaded onto the trap column (C18 PepMap100, 5 μm particle size, 300 μm × 5 mm, Thermo Fisher Scientific) for 4 minutes at 18 μL/minute; loading buffer was composed of water, 2% acetonitrile, and 0.1% TFA. Peptides were eluted with mobile phase B gradient from 4% to 35% B in 120 minutes. Eluting peptide cations were converted to gas-phase ions by electrospray ionization and analyzed using a Thermo Orbitrap Fusion instrument (Q-OT-qIT, Thermo Fisher Scientific). Survey scans of peptide precursors from 350 to 1,400 m/z were performed in Orbitrap at 120 K resolution (at 200 m/z) with a 5 × 10^5^ ion count target. Tandem MS was performed by isolation at 1.5 Th with the quadrupole, HCD fragmentation with normalized collision energy of 30, and rapid-scan MS analysis in the ion trap. The MS2 ion count target was set to 104, and the maximum injection time was 35 ms. Only precursors with charge state 2 to 6 were sampled for MS2. The dynamic exclusion duration was set to 45 seconds, with 10 ppm tolerance around the selected precursor and its isotopes. Monoisotopic precursor selection was turned on. The instrument was run at top speed mode with 2-second cycles (32).

#### Data analysis

All data were analyzed and quantified with the MaxQuant software (v2.0.3.0). The FDR was set to 1% for both proteins and peptides, and we specified a minimum peptide length of seven amino acids. The Andromeda search engine was used for the MS-MS spectra search against the Human database (downloaded from uniprot.org in January 2023 containing 20,606 entries). Enzyme specificity was set as C-terminal to arginine and lysine, also allowing cleavage at proline bonds and a maximum of two missed cleavages. Carbamidomethylation of cysteine was selected as fixed modification and N-terminal protein acetylation and methionine oxidation as variable modifications. The “match between runs” feature of MaxQuant was used to transfer identifications to other LC/MS-MS runs based on their masses and retention time (maximum deviation 0.7 minutes), and this was also used in quantification experiments. Quantifications were performed with the label-free algorithm in MaxQuant. Data analysis was performed using Perseus 1.6.15.0 software.

### Hematoxylin and eosin staining

Tumor tissue stored in 70% ethanol solution (Lach-Ner) was put into cassettes to process using an automated tissue processor (Leica ASP 6025, Leica Microsystems) according to the program standard processing overnight and embedded in paraffin blocks using the Tissue Embedding Station Leica EG1150 (Leica Microsystems). From paraffin blocks, sections of 2 μm were cut using a rotary microtome (Leica RM2255, Leica Microsystems) on standard glass slides (Waldemar Knittel, GmbH). The sections were according to a standard descriptive histopathologic staining automatically stained with hematoxylin (cat. #HHS32-1L, Sigma-Aldrich) and eosin (cat. #X883.2, Carl ROTH) and subsequently mounted using the Leica ST5020 (Leica Microsystems) automated staining instrument in combination with the Leica CV5030 coverslipper (Leica Microsystems). The microscope used was Nikon ECLIPSE 50i, and the NIS-Elements were used as the imaging software.

### Stimulated emission depletion microscopy

Cells were grown on high-performance coverslips (ZEISS) in six-well plates overnight and fixed with 4% paraformaldehyde (Sigma-Aldrich) for 15 minutes at room temperature. Samples were incubated in the permeabilization solution [0.1 mol/L glycine (Sigma-Aldrich), 0.05% Triton X-100 (Sigma-Aldrich), and 0.05% Tween-20 (Sigma-Aldrich) in PBS] for 15 minutes and blocked with 10% BSA (Sigma-Aldrich) for 30 minutes. Samples were stained overnight with primary antibodies against DNA (mouse, AC-30-10, Progene, 1:150) and Tomm20 (rabbit, ab186735, Abcam, 1:100) in the staining solution [1.5% BSA, 0.05% saponin (Sigma-Aldrich), and 0.01 mol/L glycine in PBS]. This was followed by staining with secondary antibodies abberior STAR RED (anti-mouse, 1:500) and abberior STAR 580 (anti-rabbit, 1:500) for 2 hours in the dark. The coverslips were mounted in VECTASHIELD Vibrance Antifade Mounting Medium (Vector Laboratories) and kept at 4°C until imaging. Stimulated emission depletion (STED) images were acquired on Inverted confocal microscope Nikon ECLIPSE Ti-E equipped with Nikon CFI Plan Apochromat Lambda objective (60× Oil, NA 1.40) and STED depletion laser (775 nm, 40 MHz pulsed laser, 2D donut) and processed by Fiji software.

## Results

### DHODH-dependent respiration delays acquisition of mtDNA in ρ^0^ AOX cells

**Figure 1.**
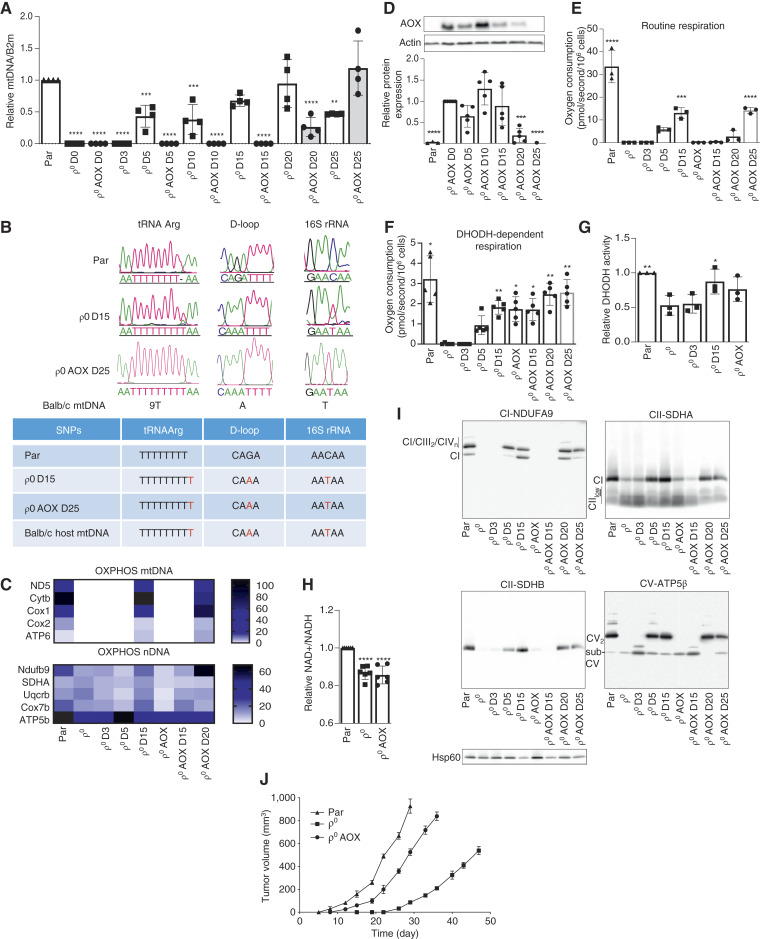
DHODH-dependent respiration delays acquisition of mtDNA in ρ^0^ cells. **A,** 4T1 ρ^0^ and 4T1 ρ^0^ AOX cells were subcutaneously injected into Balb/c mice at 10^6^ per animal. On days 3, 5, 10, 15, 20, and 25, tumor tissue was retrieved from the mice, and sublines were established. Parental cells and sublines derived from 4T1 ρ^0^ and 4T1 ρ^0^ AOX cells were assessed for mtDNA using qPCR. **B,** Parental, ρ^0^ D15, and 4T1 ρ^0^ AOX D25 cells were assessed for the distribution of mtDNA polymorphism in tRNAArg, D-loop, and 16S rRNA in single cells of lines. **C,** Heatmap illustrates mRNA expression of mitochondrial and nuclear-encoded OXPHOS genes in the cell lines indicated. The patterns were generated by the median of clustering analysis of bulk RNA sequencing data. The vertical axis represents individual samples, whereas the horizontal axis shows individual transcripts. **D,** Protein expression of AOX was assessed in parental cells, and sublines were derived from ρ^0^ AOX. **E** and **F,** Routine and DHODH-dependent respiration of parental, ρ^0^, ρ^0^ AOX, and the derived sublines was evaluated using the Oxygraph. **G** and **H,** DHODH activity was assessed in parental and ρ^0^, ρ^0^ D3, ρ^0^ D15, and ρ^0^ AOX sublines and NAD^+^/NADH ratio for parental ρ^0^, ρ^0^ AOX. **I,** Parental and ρ^0^, ρ^0^ D3, ρ^0^ D5, ρ^0^ D15, ρ^0^ AOX, ρ^0^ AOX D15, ρ^0^ AOX D20, and ρ^0^ AOX D25 cells were evaluated by native blue gel electrophoresis for the assembly of respiratory complexes and supercomplexes using antibodies to relevant subunits as shown. **J,** Balb/c mice were grafted subcutaneously with parental, ρ^0^, and ρ^0^ AOX cells (10^6^ per animal), and tumor volume was assessed on days as indicated using calipers (*n* = 3 for each group). Data are derived from at least three independent experiments. Statistical analysis was performed using ordinary one-way ANOVA and the GraphPad Prism 8 software, considering differences with the *P* value of ≤0.05 as statistically significant. *, *P* value of 0.01 to 0.05; **, *P* value of 0.001 to 0.01; ***, *P* value of 0.0001 to 0.001; ****, *P* < 0.0001.

We based the majority of our experiments on murine triple-negative breast cancer 4T1 cells, as they form syngeneic tumors in immunocompetent mice and are resistant to 6-thioguanine. More specifically, we used parental cells, their mtDNA-deficient (ρ^0^) counterparts, and ρ^0^ cells transfected with AOX (see Supplementary Fig. S1A for the appearance of the cells). In the first set of experiments, we grafted 4T1 ρ^0^ cells and their counterparts transfected with AOX into Balb/c mice and evaluated the level of mtDNA in cell lines derived from tumor tissue on different days after grafting. Figure 1A shows that although considerable amounts of mtDNA were detected in ρ^0^ cells by day 5 after grafting, and this was normalized to parental levels by day 20, ρ^0^ AOX cells only showed discernible levels of mtDNA by day 20 after grafting, i.e., after a 2-week delay. To confirm transfer of mitochondria from donor cells, we next assessed mtDNA probes that discriminate between the tRNA Arg, D-loop, and 16S rRNA polymorphism of host cells and that of 4T1 cells. We observed homoplasmic mtDNA polymorphisms of host origin in ρ^0^ D15 and ρ^0^ AOX D25 cells (Fig. 1B). To produce 4T1 ρ^0^ cells expressing AOX, we used a plasmid with the gene encoding this protein from the lower eukaryote *Ciona intestinalis* (28). Western blotting of sublines derived from pretumor plaques/tumors on different days after grafting ρ^0^ AOX cells shows that although there was similar expression of the AOX protein in ρ^0^ AOX, ρ^0^ AOX D5, ρ^0^ AOX D10, and ρ^0^ AOX D15 cells, it was lower in ρ^0^ AOX D20 cells and undetectable in ρ^0^ AOX D25 cells (Fig. 1D). This is supported at the DNA level, which shows undetectable *AOX* gene in ρ^0^ AOX D25 cells (Supplementary Fig. S1B). Thus, it can be argued that when ρ^0^ AOX cells acquire mtDNA and restore routine respiration (Fig. 1E), AOX activity and thus the AOX gene are no longer required, as routine respiration is sufficient to drive DHODH respiration/activity, and the cells therefore dispose of the “foreign” gene. In other words, *de novo* pyrimidine synthesis in ρ^0^ AOX cells in the early stages after grafting ρ^0^ AOX cells is facilitated by the oxidase.

To support these results regarding the effect of mtDNA acquisition on the mRNA level, we performed transcriptomic analysis. We selected cell lines from the period prior and after onset of mitochondrial transfer. As shown in Fig. 1C, there was no expression of transcripts of mtDNA-encoded genes in ρ^0^, ρ^0^ D3, and ρ^0^ D5 cells, despite the presence of mtDNA in D5 cells, and there was also no gene expression in ρ^0^ AOX and ρ^0^ AOX D15 cells, whereas ρ^0^ AOX D20 cells showed considerable levels of transcripts of mtDNA-encoded genes. This was confirmed using qRT-PCR, which showed that ρ^0^ and ρ^0^ AOX cells do not express mtDNA-encoded genes (Supplementary Fig. S1C). Furthermore, expression of transcripts of nuclear-encoded subunits of respiratory complexes was detected in all sublines (Fig. 1C).

We next analyzed routine and DHODH-dependent respiration in individual sublines using the specific DHODH inhibitor, leflunomide (20). Figure 1E shows that there was no routine respiration in ρ^0^ and ρ^0^ D3 cells, although it was partially restored in ρ^0^ D5 and more so in ρ^0^ D15 cells, whereas there was no routine respiration in ρ^0^ AOX cells until day 20, in which slight respiration recovery was observed, followed by higher routine respiration in ρ^0^ D25 AOX cells (Fig. 1E). Restoration of DHODH-dependent respiration, which is essential for the conversion of dihydroorotate to orotate catalyzed by DHODH, followed a similar pattern in sublines derived from tumors grown from ρ^0^ cells. However, this was in stark contrast to cells derived from ρ^0^ AOX tumors that showed DHODH-dependent respiration in all sublines (Fig. 1F). In accord with this, the presence of AOX in ρ^0^ cells resulted in increased proliferation in the absence of uridine in comparison with ρ^0^ cells (Supplementary Fig. S1D), albeit at a much lower rate than seen for parental cells. Sublines derived from tumors grown from ρ^0^ cells proliferated according to the level of restored mitochondrial respiration and the extent of acquired mtDNA (Supplementary Fig. S1D). However, DHODH activity was only modestly altered, in comparison with DHODH-dependent respiration (Fig. 1G), the reason being that DHODH activity assay is based on endogenously added substrate, which is not dependent on CoQ redox cycling, unlike DHODH-dependent respiration (20). However, the absence of routine respiration was evident in the decreased NAD^+^/NADH ratio observed in both ρ^0^ and ρ^0^ AOX cells (Fig. 1H). To further characterize these sublines, we evaluated the ratio of ATP generated by OXPHOS and by glycolysis. Although it was about 1:1 in parental cells and in ρ^0^ D5 and ρ^0^ D15 cells, it was shifted toward glycolysis in ρ^0^ and ρ^0^ D3 cells and in ρ^0^ AOX cells (Supplementary Fig. S1E). This follows the pattern of assembly of respiratory complexes and supercomplexes, which were not assembled in ρ^0^, ρ^0^ D3, ρ^0^ AOX, and ρ^0^ AOX D15 cells (Fig. 1I), consistent with the finding that these cell lines showed little or no routine respiration, whereas complexes were assembled in ρ^0^ D5 and ρ^0^ D15 cells and in AOX ρ^0^ D20 and AOX ρ^0^ D25 cells, again showing a 2-week delay for the latter (c.f. Fig. 1E).

Finally, to investigate the role of AOX-powered DHODH-dependent respiration in tumor growth from ρ^0^ cells, we grafted parental ρ^0^ and ρ^0^ AOX cells into Balb/c mice and followed tumor volume over time. Although we observed a considerable delay of about 21 days in tumor growth with ρ^0^ cells, ρ^0^ AOX cells formed tumors with only a small delay of about 4 days compared with parental cells (Fig. 1J), reinforcing previous data (20).

These results clearly demonstrate that cells devoid of mtDNA and therefore lacking DHODH-dependent respiration need to acquire mitochondria for recovery of DHODH-dependent respiration, which is necessary for *de novo* pyrimidine synthesis and hence tumor cell proliferation. On the other hand, ρ^0^ cells expressing the AOX protein are able to proliferate, as they maintain DHODH-dependent respiration needed for *de novo* pyrimidine synthesis and therefore are not under the same immediate pressure to acquire mitochondria from the stroma after grafting, unlike their ρ^0^ counterparts.

### Absence of DHODH suppresses efficient tumor growth

**Figure 2.**
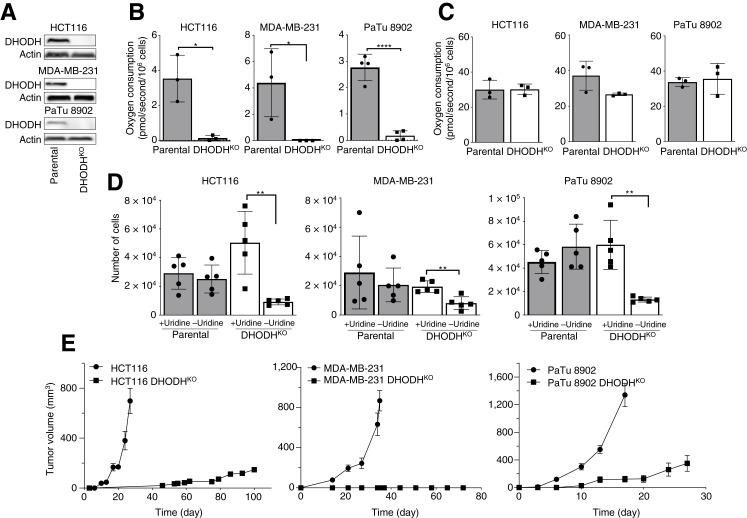
Absence of DHODH suppresses efficient tumor growth. **A,** Expression of DHODH in parental and DHODH^KO^ cells (HCT116, MDA-MB-231, and PaTu 8902 cell lines) was assessed by Western blotting analysis. **B–D,** Routine and DHODH-dependent respiration was evaluated in parental and DHODH^KO^ cells (**B** and **C**), as well as their proliferation (number of cells; **D**) at 72 hours in the presence and absence of uridine in the media. **E,** NSG mice were grafted subcutaneously with parental and DHODH^KO^ cells (10^6^ per animal), and tumor volume was assessed using calipers (*n* = 6 for each group). The experiments are representative of at least three independent repetitions (*n* ≥ 3). Statistical analysis was performed using an unpaired *t* test with GraphPad Prism 8 software, considering differences with a *P* value of ≤ 0.05 as statistically significant. Images are representative of three independent experiments of three separate biological replicates. *, *P* value of 0.01 to 0.05; **, *P* value of 0.001 to 0.01.

We next queried whether DHODH is required for tumor growth across a range of human cancer cells of different origin, which we have shown earlier for mouse breast cancer 4T1 cells (20). First, we prepared DHODH-deficient (DHODH^KO^) cells of colorectal cancer (HCT116), breast cancer (MDA-MB-231), and pancreatic cancer origin (PaTu 8902; Fig. 2A). We found that these cells did not show DHODH-dependent respiration (Fig. 2B), although there was no significant effect on routine respiration (Fig. 2C). We also found that DHODH^KO^ cells proliferated similarly to parental cells only in the presence of uridine in the media (Fig. 2D), which points to the role of the salvage pathway of pyrimidine synthesis in the growth of DHODH^KO^ cells. We then grafted parental cells and their DHODH^KO^ counterparts into immunocompromised NSG mice and monitored the kinetics of tumor growth and progression. Figure 2E shows that tumor growth occurred at an early stage and with rapid kinetics for respiration-competent parental cells, with functional DHODH-dependent respiration component. On the other hand, DHODH^KO^ cells either did not form tumors at all (MDA-MB-231 DHODH^KO^ cells), or small tumors appeared at a later stage and showed very slow growth kinetics (HCT116 DHODH^KO^ and PaTu 8902 DHODH^KO^ cells).

These data confirm that DHODH is required for efficient onset of tumor growth, with little or no progression in its absence, and that routine respiration maintained in DHODH^KO^ cells does not suffice to drive early tumor growth.

### Deficiency of mtDNA results in early recruitment of protumorigenic immune cells

HMT, epitomized here by mtDNA acquisition, is needed for the restoration of the respiratory function required for driving *de novo* pyrimidine synthesis via DHODH. In order to import mitochondria with mtDNA, cancer cells with severe defects in their mitochondrial genome need to recruit potential donor cells from the stroma. To study the composition of stroma, including the presence of immune cells, we used mouse breast tumor cells (4T1, 4T1 ρ^0^, and 4T1 DHODH^KO^) that form syngeneic tumors in immunocompetent animals. We queried whether the need for acquisition of mtDNA by HMT in 4T1ρ^0^ cells relative to 4T1 cells, i.e., cells with dysfunctional and functional *de novo* pyrimidine synthesis, respectively, is linked to differences in the early establishment and composition of tumor stroma and whether a different model with dysfunctional pyrimidine synthesis, 4T1 DHODH^KO^, has the same effect on cellular composition of the tumor microenvironment. We grafted 4T1, 4T1 ρ^0^, and 4T1 DHODH^KO^ cells into Balb/c mice and evaluated the cellular composition of tumor stroma on days 3, 5, and 15 in each cell line and on day 40 in 4T1 ρ^0^ cells using FCM and specific staining for different subpopulations of immune cells (see Supplementary Fig. S2 for gating strategy). Using hematoxylin and eosin staining, we also assessed the appearance of tumors derived from 4T1, 4T1 ρ^0^, and 4T1 DHODH^KO^ cells on D3 after grafting (Supplementary Fig. S4).

**Figure 3.**
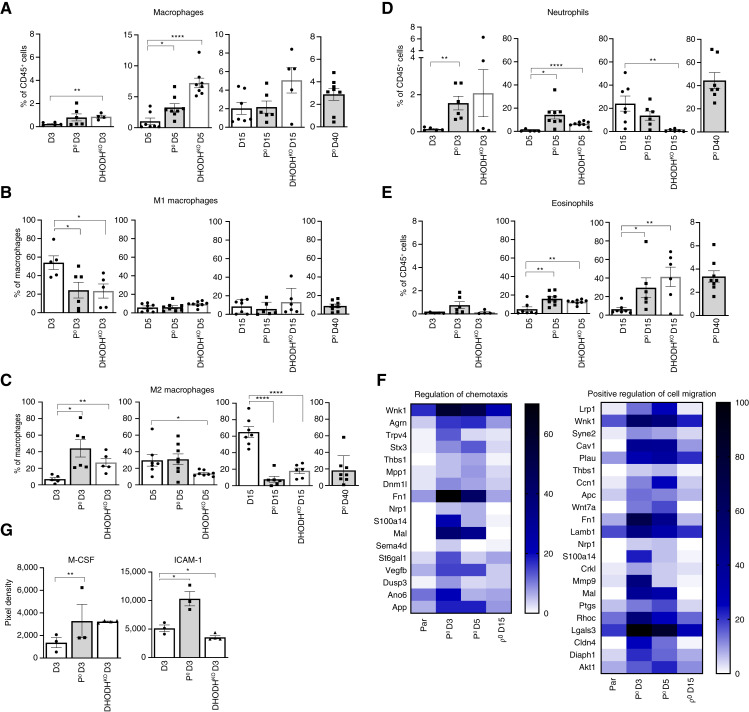
Deficiency of mtDNA results in early recruitment of protumor immune cells. **A–E,** Balb/c mice were injected subcutaneously with parental, 4T1 ρ^0^, and 4T1 DHODH^KO^ cells (10^6^ per animal), and tumor tissue was assessed for the percentage of macrophages, M1 and M2 macrophages, neutrophils, and eosinophils by FCM using a panel of mAb at the time points shown. The 4T1 ρ^0^ and 4T1 DHODH^KO^ samples were comprised of 2 to 4 tumors due to their small size, whereas parental samples represented individual tumors from distinct animals. **F,** The heatmap illustrates the level of transcripts of genes regulating chemotaxis and cell migration in parental, ρ^0^, ρ^0^ D3, ρ^0^ D5, and ρ^0^ D15 cells after 72 hours of cultivation without uridine. **G,** Balb/c mice were grafted with cells shown as per above, and their serum was assessed for M-CSF and ICAM-1. The data shown are median values with SEM for groups, each comprising at least 5 samples per group for a specific time point. Statistical significance between groups was assessed using an unpaired *t* test through GraphPad Prism 8 software. Differences with *P* value ≤ 0.05 were considered statistically significant. *, *P* value of 0.01 to 0.05; **, *P* value of 0.001 to 0.01; ****, *P* < 0.0001.

In the early phase of tumor progression (D3 and D5), in grafted ρ^0^ and 4T1 DHODH^KO^ cells, we observed considerable differences in immune cell infiltration, including macrophages (Fig. 3A–C), neutrophils (Fig. 3D), and eosinophils (Fig. 3E). M2 macrophages and neutrophils are well known for their protumor activity and poor patient prognosis (33–37), whereas the protumor effect of eosinophils seems to be more cancer tissue–specific (38, 39), and include modulation of the tumor microenviroment (40). Additionally, research by Michaeli and colleagues has shown that neutrophils in the tumor microenvironment can induce apoptosis of nonactivated CD8 T cells, further highlighting their role in supporting tumor progression (41). To this end, there was a higher percentage of M2 protumor macrophages infiltrating the stroma on day 3 after grafting in ρ^0^ cells and 4T1 DHODH^KO^ cells compared with parental cells (Fig. 3C), whereas the opposite was observed for M1 macrophages (Fig. 3B). Differences in infiltration of protumor immune cells change with time across the three models. On day 15, infiltration of M2 macrophages and neutrophils increased in parental tumors, indicating that at this stage parental tumors may need a more protective environment (Fig. 3C and D). Eosinophils may serve longer in creating a protective environment, so they are still significantly increased in stroma of ρ^0^ cells on day 15 compared with parental cells (Fig. 3E). The reason for this may be due to the specific protumor activity of eosinophils such as modulation of the microenvironment through the production of growth factors, which aid in tumor cell proliferation or activation of angiogenesis (40). We additionally measured the infiltration of monocytes, B lymphocytes, and T lymphocytes (Supplementary Fig. S3A–S3D), and we observed a negative correlation between neutrophils and T cells (CD4^+^ and CD8^+^) in the tumor microenvironment following ρ^0^ cell engraftment and observed no correlation with regulatory T cells. These findings suggest that cells with inactive pyrimidine synthesis attract neutrophils to the tumor microenvironment, thereby inhibiting the effect of antitumor CD8^+^ and CD4^+^ T cells and promoting a protumorigenic environment (Supplementary Fig. S3E).

We then evaluated parental cells and cells derived from tumors formed from ρ^0^ cells on days 3, 5, and 15 after grafting for expression of genes regulating chemotaxis and cell migration (Fig. 3F). Interestingly, we found that although there was low expression of these genes in parental cells, they were highly expressed in ρ^0^ cells and in ρ^0^ D3 and ρ^0^ D5 cells, whereas their level was low in ρ^0^ D15 cells that already show increased level of respiration following acquisition of mtDNA from the stroma (cf. Fig. 1A, E, and F). This indicates that cancer cells lacking respiration have the propensity to attract cells of the immune system early after grafting, presumably in order to establish a protumor environment permissive for tumor progression prior to HMT.

Additionally, on day 3, we detected a significantly higher level of ICAM-1 in the serum of mice injected with ρ^0^ cells and a significant increase in M-CSF in mice injected with ρ^0^ cells or DHODH^KO^ cells (Fig. 3G). It has been demonstrated that M-CSF acts as a chemoattractant for macrophages to the tumor microenvironment, and M-CSF receptor also regulates differentiation of macrophages into the tumor-promoting M2 phenotype (42). It is also known that when ICAM-1 is inhibited, neutrophils cannot penetrate the endothelial barrier, compromising their entry into the tumor (43). Additionally, soluble ICAM-1 plays a role in the recruitment of macrophages to glioblastomas (44).

### MSCs are recruited early after grafting ρ^0^ cells

**Figure 4.**
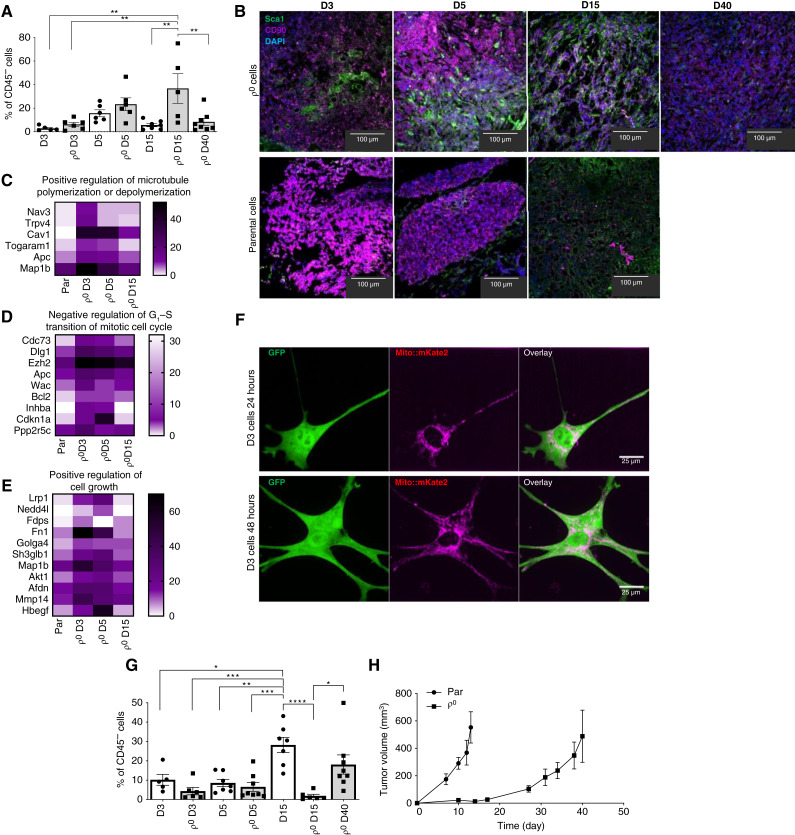
MSCs are recruited early after grafting ρ^0^ cells. **A,** Tumor tissue derived from parental and ρ^0^ cells, as shown, was evaluated by FCM for MSCs as a percentage of nonimmune, CD45^−^ cells, as cells positive for Sca1 and CD90. **B,** Tumor tissue derived from parental and ρ^0^ cells on days shown was evaluated by confocal microscopy for Sca1^+^ and CD90^+^ cells following staining with relevant antibodies. DAPI was used for the detection of nuclei. **C–E,** Heatmap illustrating diverse mRNA expression of positive regulation of microtubule polymerization or depolymerization, negative regulation of G_1_–S transition of the mitotic cell cycle, and positive regulation of cell growth in parental, ρ^0^, ρ^0^ D3, ρ^0^ D5, and ρ^0^ D15 cells after 72-hour cultivation without uridine. Patterns were generated through clustering analysis of bulk RNA sequencing data. The vertical axis represents individual samples, whereas the horizontal axis delineates the various mRNA transcripts. **F,** MSCs isolated from mito::mKate2 mice were combined with GFP-positive ρ^0^ cells at 1:3 and grafted in Geltrex in Balb/c mice. After 3 days, mice were sacrificed. The tissue derived from the grafted cell mixture was placed in culture flasks, and 24 and 48 hours later, the cells were inspected by confocal microscopy for red and green fluorescence. **G,** Parental and ρ^0^ cells were grafted in Balb/c mice (10^6^ per animal), and the tumor tissue was evaluated by FCM for the presence of cancer cells in the stroma, as the percentage of CD24^+^/CD49f^+^ (breast cancer) cells of CD45^−^ (nonimmune) cells. **H,** Tumor volume was evaluated using calipers. Six mice were used for each group. The presented data reflect mean values with SEM for groups. *, *P* value of 0.01 to 0.05; **, *P* value of 0.001 to 0.01; ***, *P* value of 0.0001 to 0.001; ****, *P* < 0.0001.

As MSCs are the most promising mitochondrial donor candidates (4, 18, 45, 46), we assessed their presence in the tumor microenvironment of ρ^0^ cells on days 3, 5, 15, and 40 (ρ^0^ cells only) by FCM. MSCs were detected as cells positive for Sca1 and CD90, as a percentage of nonimmune, CD45^−^ cells. Figure 4A shows increased MSCs as a percentage of CD45 cells in tumors derived from ρ^0^ cells on days 3, 5, and 15, i.e., during the early stages of tumor progression, although their level was low in ρ^0^ D40 cells. On the other hand, MSCs were consistently lower in stroma of tumors derived from parental cells. This pattern was confirmed by confocal microscopy of tumor sections using anti-Sca1 and CD90 IgGs, in which the presence of white color (overlay of red and green fluorescence) indicates cells positive for both markers (Fig. 4B). These results show that ρ^0^ cells, compared with their parental counterparts, recruit MSCs at a higher level, consistent with these cells being mitochondrial donors early after grafting.

To further investigate MSCs as donors of mitochondria for ρ^0^ cells, we used an *in vivo* coculture system based on subcutaneous grafting of a mixture of MSCs derived from mito::mKate2 mice with mitochondria decorated with far-red fluorescent protein mKate2 and 4T1 ρ^0^ cells expressing cytosolic GFP. Tumors derived from ρ^0^ and parental cells were excised on days 3, 5, and 15, and tumor tissue was placed in culture dishes. Cancer cells positive for GFP growing from the tumor were then inspected for the presence of red mitochondria. Figure 4F shows that there was a high number of mitochondria with red fluorescence in breast cancer cells grown from tumor tissue derived from ρ^0^ cells. On the other hand, no transfer was observed in coculture of parental 4T1 cytosolic GFP cells with mito::mKate2 MSCs (Supplementary Fig. S5A). As MSCs have been proposed to transfer mitochondria to ρ^0^ cells via TNTs containing tubulin fibers as mitochondrial conduits (5, 13), we assessed parental, ρ^0^, ρ^0^ D3, ρ^0^ D5, and ρ^0^ cells D15 cells for expression of genes involved in remodeling of tubulin and found that although there was low level expression of these genes in parental cells, it was high in ρ^0^ cells and cells derived from tumors grown from ρ^0^ cells on different days (Fig. 4C). These results support the view that MSCs are donors of mitochondria for grafted ρ^0^ cells, most likely using TNTs as conduits of mitochondrial transfer.

We next queried how the initial lack of mtDNA and respiration that drives *de novo* pyrimidine synthesis affects tumor progression. Therefore, we subjected tumor samples to transcriptomic analysis to investigate expression of genes involved in negative regulation of the G_1_–S transition of the mitotic cell cycle (Fig. 4D) and positive regulation of cell growth (Fig. 4E). The results showed increased expression of these genes in ρ^0^ D3 and ρ^0^ D5 cells (compared with parental cells), consistent with G_1_–S arrest of cellular proliferation of ρ^0^ cells early after grafting.

We showed earlier that ρ^0^ cells need to acquire mitochondria to restore DHODH-dependent respiration, leading to recovery of *de novo* pyrimidine synthesis and hence cellular proliferation and tumor growth. We therefore used imaging by STED microscopy to assess the presence of mtDNA in parental and ρ^0^ cells and cells derived from tumors grown from ρ^0^ cells 3, 5, and 15 days after grafting. Supplementary Figure S5B shows a high level of mitochondrial nucleoids in parental cells and their absence in ρ^0^ and ρ^0^ D3 cells, although their number was low in ρ^0^ D5 cells and higher in ρ^0^ D15 cells. Quantitation of the images in Supplementary Fig. S5C shows a low number of nucleoids per 1 μm^2^ of mitochondria, although this number is higher for ρ^0^ D15 cells.

To link the above results on early infiltration of stroma derived from grafted ρ^0^ cells compared with parental cells, by immune cells and MSCs that provide a protumor environment and a source of mitochondrial transfer donors and subsequent tumor growth, we evaluated formation and progression of tumors derived from parental and ρ^0^ cells. We first analyzed the tumor tissue, including its stroma, for the number of cancer cells, expressing CD24^+^/CD47f^+^ cells as a percentage of CD45^−^ nonimmune cells. Figure 4G shows that the number of breast cancer cells within the stroma is lower for ρ^0^ cells compared with parental cells on days 3, 5, and 15 after grafting and is relatively high in stroma derived from ρ^0^ cells on day 40 after grafting. This finding is supported by previously mentioned data from transcriptomics (Fig. 4C) and importantly by the kinetics of tumor growth by parental and ρ^0^ cells, showing approximately 3-week delay for the ρ^0^ cell–derived tumors to develop kinetics similar to that of tumors derived from parental cells (Fig. 4H).

These data link HMT with high early infiltration of tumor stroma with protumor immune cells and MSCs and with restoration of proliferation of the original ρ^0^ cells following grafting in the context of onset of tumor growth.

## Discussion

Recent research has shown that cancer cells with defects in mtDNA acquire mitochondria with their mtDNA payload from cells in the stroma in order to restore mitochondrial function (4, 6, 19, 20, 46–48). One aspect of this recovery concerns mitochondrial respiration, a process that is inherent to mitochondria and critical for a number of functions, including mitochondrial ATP formation. We showed that cancer cells with dysfunctional mitochondria due to mtDNA deficiency acquire mitochondria with mtDNA by HMT from the tumor stroma to restore *de novo* pyrimidine synthesis rather than for the purpose of efficient ATP production (20). In fact, cancer cells that do not respire can produce enough ATP by glycolysis when sufficient glucose is available (20, 21). This is a consequence of the high metabolic “plasticity” of cancer cells, i.e., their ability to rapidly adjust their mode of energy generation and metabolic regulation to specific conditions that are often unfavorable (49–52). For example, we found that alternative assembly of mitochondrial complexes (epitomized by CII) helps cancer cells survive under energetically challenging conditions (53).

It seems that of the ETC components, CIII and CIV are essential to drive *de novo* pyrimidine synthesis, whereas other complexes (CI and CII) are less important, if not redundant, for the onset of tumor growth. This is due to the function of CIII and CIV, which includes redox cycling of CoQ (20, 54, 55) so that it can accept electrons from DHODH, critical for *de novo* pyrimidine synthesis in mitochondria (20, 23, 56). We performed experiments to determine whether DHODH-dependent respiration, required for DHODH enzyme activity, is important for the initiation of tumor growth, as this has implications for both basic and translational science. Our major tools to obtain initial insight into this phenomenon were 4T1 ρ^0^ cells and their counterparts transfected with AOX from *Ciona intestinalis* (57). The reason for this was to use a cell line that lacks mitochondrial respiration/OXPHOS (due to the absence of mtDNA that encodes 13 subunits of CI, CIII, CIV, and CV) but is transfected with AOX with functions of CIII plus CIV, enabling acceptance of electrons by CoQ that then drives DHODH and *de novo* pyrimidine synthesis (20, 27, 57). We grafted these cell lines into immunocompetent mice and, interestingly, found that although the presence of mtDNA of host origin was already evident 5 days after grafting of ρ^0^ cells, this was delayed by additional 15 days for ρ^0^ AOX cells, as we detected mtDNA of host origin by D20 in ρ^0^ AOX cells. The presence of AOX with its capacity to reoxidize ubiquinol (20, 54, 55) enabled early tumor growth and delayed mtDNA acquisition.

We further characterized the sublines derived from pretumor plaques/small tumors grown from 4T1 ρ^0^ and 4T1 ρ^0^ AOX cells and found that delayed tumor growth in ρ^0^ AOX cells also affected other functions, such as recovery of routine respiration, whereas DHODH-dependent respiration was present in all ρ^0^ AOX sublines. On the other hand, DHODH-dependent respiration was detected in ρ^0^ sublines only after mitochondrial acquisition and recovery of routine respiration, which correlated with the assembly of mitochondrial complexes. The assembly of respiratory complexes in ρ^0^ AOX cells did not have a consistent effect on DHODH-dependent respiration. Interestingly, we found that ρ^0^ AOX sublines isolated from small tumors 20 and 25 days after grafting showed low or no expression of the AOX protein and loss of the *AOX* gene, coinciding with the assembly of mitochondrial complexes and recovery of routine respiration. This strongly indicates that when no longer needed, cells lost the AOX gene expression, possibly by epigenetic silencing or DNA recombination (58, 59).

The above data show that ρ^0^ cells expressing AOX, which drives DHODH-dependent respiration, delay the early onset of mtDNA acquisition via HMT, suggesting that DHODH and associated *de novo* pyrimidine synthesis are important for the onset of tumor growth (20, 60–63). To directly verify the relevance of this finding, we evaluated human cancer cells of different origin for their efficacy to form tumors and compared this with their DHODH^KO^ counterparts, including colorectal, breast, and pancreatic cancer cells. We observed fast tumor growth after grafting of parental cells, whereas the DHODH^KO^ cells either did not form tumors at all (MDA-MB-231 breast cancer cells) or formed tumors with considerable delay and slow kinetics. These results clearly point to the importance of DHODH (and associated *de novo* pyrimidine synthesis) for the early onset of efficient tumor growth. As the DHODH status cannot be rectified after grafting of DHODH^KO^ cells, the fact that tumor growth occurred in the case of colorectal and pancreatic tumors, albeit delayed and slow, suggests a minor role for salvage pathways in these tumor models. Although *de novo* pyrimidine synthesis is energetically highly demanding, the more economical salvage pathways of pyrimidine, as well as purine generation, are dependent on specific substrates (often products of DNA and RNA degradation), which may be dictated by the specific tumor environment (64, 65). From a clinical viewpoint, targeting the *de novo* pyrimidine biosynthetic pathway by means of DHODH inhibition is considered a potential cancer therapy that could be applied across a wide spectrum of tumors (23, 24, 49, 52, 66).

The finding that cells with compromised mitochondrial respiratory function need to acquire mtDNA to restore respiration in order to drive *de novo* pyrimidine synthesis, whereas respiratory-competent cells readily form tumors (4, 20), suggests a possible difference in the composition of the microenvironment of the stroma established following tumor cell grafting. This includes, in particular, the cellular composition of the stroma with focus on immune cells that promote or suppress tumor progression (60–62). Although we used immunocompromised mice to investigate the effect of DHODH deficiency on the ability of human cancer cells to form tumors, we used immunocompetent mice to investigate possible differences in the cellular composition of stroma in syngeneic tumors derived from 4T1 and 4T1 ρ^0^ cells. Of the immune cells evaluated in the tumor stroma, we observed an increase in M2 macrophages, eosinophils, and neutrophils soon after grafting ρ^0^ cells compared with their respiratory-competent counterparts, whereas the opposite was the case after a longer period of grafting. Interestingly, although the number of the antitumor M1 macrophages was lower for ρ^0^ cells shortly after their grafting compared with 4T1 cells, the number of the protumor M2 macrophages was much higher. These results suggest that cells with compromised mitochondrial respiration that cannot form tumors readily, as they need to import mitochondria with mtDNA from the stroma, attract cells of the immune system that provide a protective, tumor-permissive environment until the point when they are ready to start efficiently forming tumors (67–70).

Finally, we queried whether cancer cells with compromised respiration attract cells that are potential mitochondrial donors to promote tumor growth. Although in the case of brain tumors such as glioblastomas, astrocytes are considered the major source of mitochondria for respiration-dysfunctional tumor cells (47, 71), in most other solid tumors, MSCs are thought to be prime candidates for mitochondrial donation (46–48, 70). In this study, using *in vivo* coculture of ρ^0^ cells and MSCs, we show their ability to act as effective mitochondrial donors. Moreover, we also observed differences in the number of MSCs in the stroma of small tumors derived from parental 4T1 cells and their ρ^0^ counterparts. Consistent with the notion that ρ^0^ cells acquire mtDNA by way of transfer of whole mitochondria (19), we observed significantly higher MSCs in early tumor stroma after grafting ρ^0^ cells compared with respiration-competent parental cells. This reflects the delay involving acquisition of mitochondria by ρ^0^ cells with subsequent tumor growth. This ‘lag time’ is needed for the cells to restore functional mitochondria and DHODH-dependent respiration, leading to recovery of *de novo* pyrimidine synthesis and hence cellular proliferation and tumor growth.

Although we assume that the major role of DHODH in cancer cells is linked to powering *de novo* pyrimidine synthesis, which is the major focus of this research, it is likely that there is yet another layer of its contribution to tumor growth. A very recent study has shown that DHODH modulates immune surveillance, in particular cytotoxicity of CD8 T cells (72). This is reminiscent of our results showing differences in the recruitment of immune cells to the stroma in relation to the DHODH status of grafted cancer cells. This strongly indicates that the role of DHODH in tumor onset and progression is multifaceted, pointing to it as a target for cancer therapy, in particular when combined with immunotherapy presented by immune checkpoint inhibitors (72).

To conclude, we present data that unequivocally point to the crucial importance of respiration driving *de novo* generation of pyrimidines for rapid kinetics of tumor growth, which is supported by early recruitment of mitochondria from donor cells into the microenvironment. We propose that targeting *de novo* pyrimidine synthesis at the level of DHODH presents a widely applicable approach to cancer therapy, alone or in combination with other anticancer agents with a different mode of action. Moreover, inhibition of mitochondrial transfer and acquisition by tumor cells with impaired mitochondrial respiration, which can be a result of certain therapeutic regimens, could increase the effectiveness of anticancer treatment directed at mitochondrial components and may ideally counteract the possible development of resistance to therapy. This is in line with the hypothesis that mitochondria increase cancer cell “fitness”, consistent with the theory of “survival of the fittest” (73, 74). In other words, curbing (mitochondrial) fitness of tumor cells may help treat cancer.

## Supplementary Material

Figure S1 Properties related to parental, rho0 and rho0 AOX cells

Figure S2 Gating strategy for flow cytometry

Figure S3 Immune cells in tumor stroma

Figure S4 H&E staining

Figure S5 Mitochondrial nucleoids

Table S1 Flow cytometry antibodies

## Data Availability

RNA sequencing data generated in this study are available in ArrayExpress at accession number E-MTAB-15669 (https://www.ebi.ac.uk/biostudies/ArrayExpress/studies/E-MTAB-15669?key=a9510c82-6089-438f-a010-1849c2823308), and mtDNA sequencing data are available in Zenodo (https://zenodo.org/records/17285413). All other raw data are available upon request to the corresponding author.
