# Supplementary material for: Mitochondrial Transfer Rescues Respiration to Support De Novo Pyrimidine Biosynthesis and Tumor Progression
Source: Cancer Res. 2025 Nov 17;86(4):925–39. doi: 10.1158/0008-5472.CAN-24-0737 (PMC13053058; doi:10.1158/0008-5472.CAN-24-0737)
Supplement: Figure S1 — Properties related to parental, rho0 and rho0 AOX cells [file can-24-0737_figure_s1_suppsf1.pptx]

## Slide 1
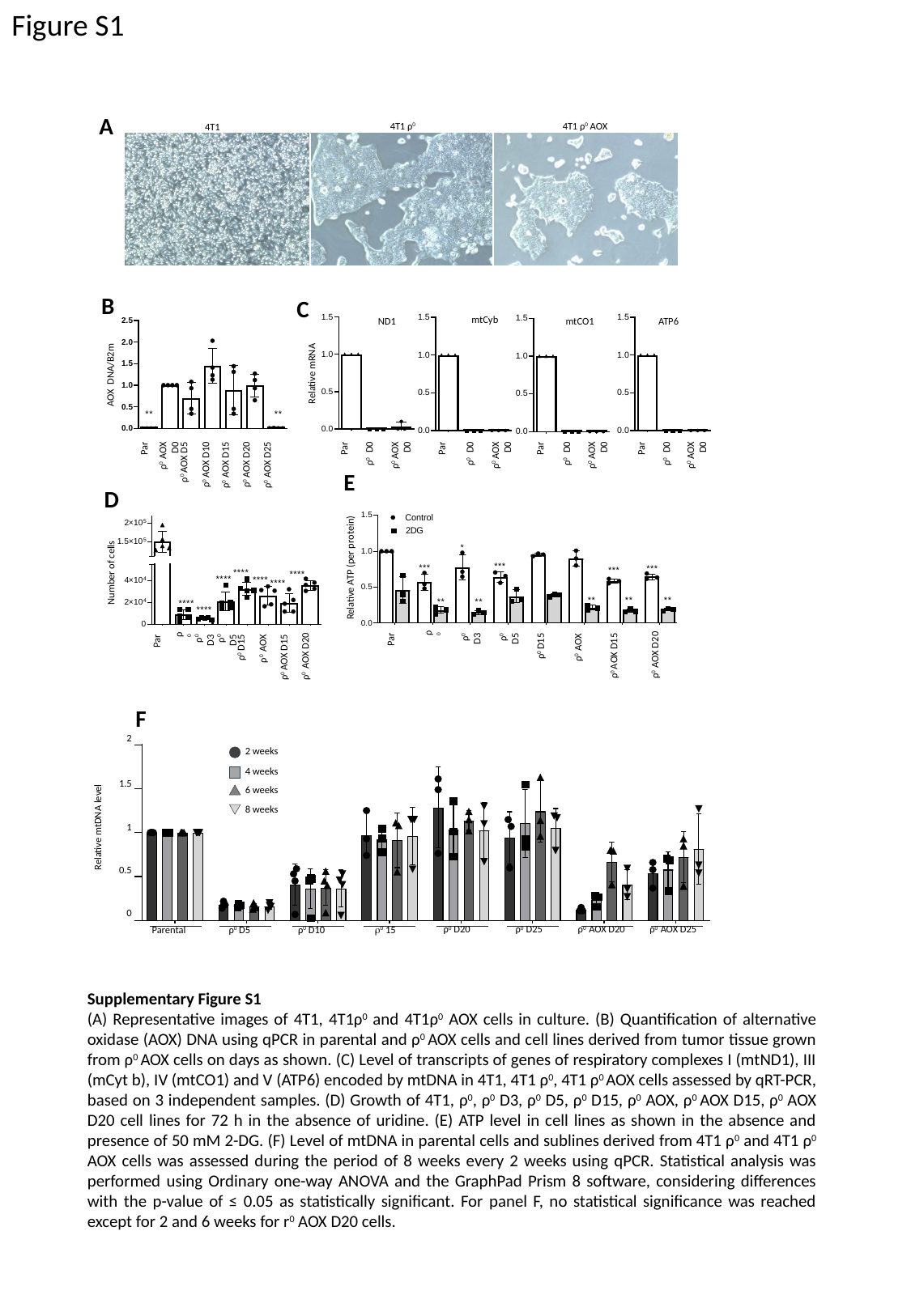

Figure S1
A
4T1 ρ0 AOX
4T1 ρ0
4T1
B
AOX DNA/B2m
Par
ρ0 AOX D0
ρ0 AOX D5
ρ0 AOX D10
ρ0 AOX D15
ρ0 AOX D20
ρ0 AOX D25
C
mtCyb
ND1
mtCO1
ATP6
Relative mRNA
Par
ρ0 D0
ρ0 AOX D0
Par
ρ0 D0
ρ0 AOX D0
Par
ρ0 D0
ρ0 AOX D0
Par
ρ0 D0
ρ0 AOX D0
E
D
Relative ATP (per protein)
Number of cells
ρ0 AOX D20
Par
ρ0
ρ0 D3
ρ0 D5
ρ0 D15
ρ0 AOX
ρ0 AOX D15
ρ0 AOX D20
Par
ρ0
ρ0 D3
ρ0 D5
ρ0 D15
ρ0 AOX
ρ0 AOX D15
F
2
2 weeks
4 weeks
1.5
6 weeks
8 weeks
Relative mtDNA level
1
0.5
ρ0 AOX D20
ρ0 AOX D25
0
Parental
ρ0 D20
ρ0 D25
ρ0 D10
ρ0 D5
r0 15
Supplementary Figure S1
(A) Representative images of 4T1, 4T1ρ0 and 4T1ρ0 AOX cells in culture. (B) Quantification of alternative oxidase (AOX) DNA using qPCR in parental and ρ0 AOX cells and cell lines derived from tumor tissue grown from ρ0 AOX cells on days as shown. (C) Level of transcripts of genes of respiratory complexes I (mtND1), III (mCyt b), IV (mtCO1) and V (ATP6) encoded by mtDNA in 4T1, 4T1 ρ0, 4T1 ρ0 AOX cells assessed by qRT-PCR, based on 3 independent samples. (D) Growth of 4T1, ρ0, ρ0 D3, ρ0 D5, ρ0 D15, ρ0 AOX, ρ0 AOX D15, ρ0 AOX D20 cell lines for 72 h in the absence of uridine. (E) ATP level in cell lines as shown in the absence and presence of 50 mM 2-DG. (F) Level of mtDNA in parental cells and sublines derived from 4T1 ρ0 and 4T1 ρ0 AOX cells was assessed during the period of 8 weeks every 2 weeks using qPCR. Statistical analysis was performed using Ordinary one-way ANOVA and the GraphPad Prism 8 software, considering differences with the p-value of ≤ 0.05 as statistically significant. For panel F, no statistical significance was reached except for 2 and 6 weeks for r0 AOX D20 cells.
