# Supplementary material for: Mitochondrial Transfer Rescues Respiration to Support De Novo Pyrimidine Biosynthesis and Tumor Progression
Source: Cancer Res. 2025 Nov 17;86(4):925–39. doi: 10.1158/0008-5472.CAN-24-0737 (PMC13053058; doi:10.1158/0008-5472.CAN-24-0737)
Supplement: Figure S2 — Gating strategy for flow cytometry [file can-24-0737_figure_s2_suppsf2.pptx]

## Slide 1
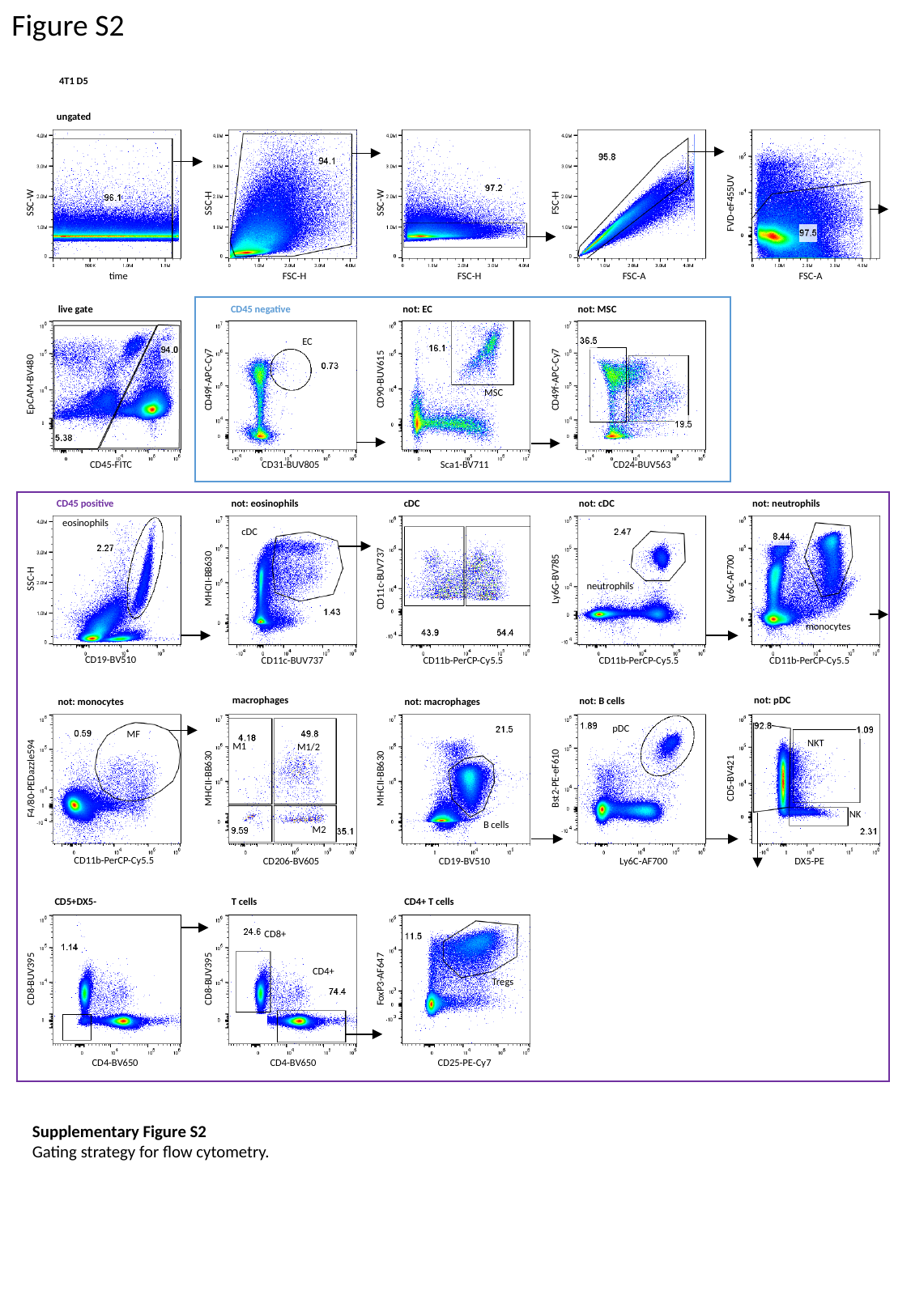

Figure S2
4T1 D5
ungated
SSC-W
SSC-H
SSC-W
FSC-H
FVD-eF455UV
FSC-H
FSC-A
FSC-A
time
FSC-H
live gate
CD45 negative
not: EC
not: MSC
EC
CD49f-APC-Cy7
CD90-BUV615
CD49f-APC-Cy7
EpCAM-BV480
MSC
Sca1-BV711
CD24-BUV563
CD45-FITC
CD31-BUV805
CD45 positive
not: eosinophils
cDC
not: cDC
not: neutrophils
eosinophils
cDC
SSC-H
MHCII-BB630
CD11c-BUV737
Ly6G-BV785
Ly6C-AF700
neutrophils
monocytes
CD19-BV510
CD11c-BUV737
CD11b-PerCP-Cy5.5
CD11b-PerCP-Cy5.5
CD11b-PerCP-Cy5.5
macrophages
not: pDC
not: B cells
not: macrophages
not: monocytes
pDC
MF
NKT
M1
M1/2
F4/80-PEDazzle594
MHCII-BB630
MHCII-BB630
Bst2-PE-eF610
CD5-BV421
NK
B cells
M2
CD11b-PerCP-Cy5.5
Ly6C-AF700
DX5-PE
CD206-BV605
CD19-BV510
CD5+DX5-
T cells
CD4+ T cells
CD8+
CD4+
CD8-BUV395
CD8-BUV395
FoxP3-AF647
Tregs
CD25-PE-Cy7
CD4-BV650
CD4-BV650
Supplementary Figure S2
Gating strategy for flow cytometry.
