# Supplementary material for: Mitochondrial Transfer Rescues Respiration to Support De Novo Pyrimidine Biosynthesis and Tumor Progression
Source: Cancer Res. 2025 Nov 17;86(4):925–39. doi: 10.1158/0008-5472.CAN-24-0737 (PMC13053058; doi:10.1158/0008-5472.CAN-24-0737)
Supplement: Figure S3 — Immune cells in tumor stroma [file can-24-0737_figure_s3_suppsf3.pptx]

## Slide 1
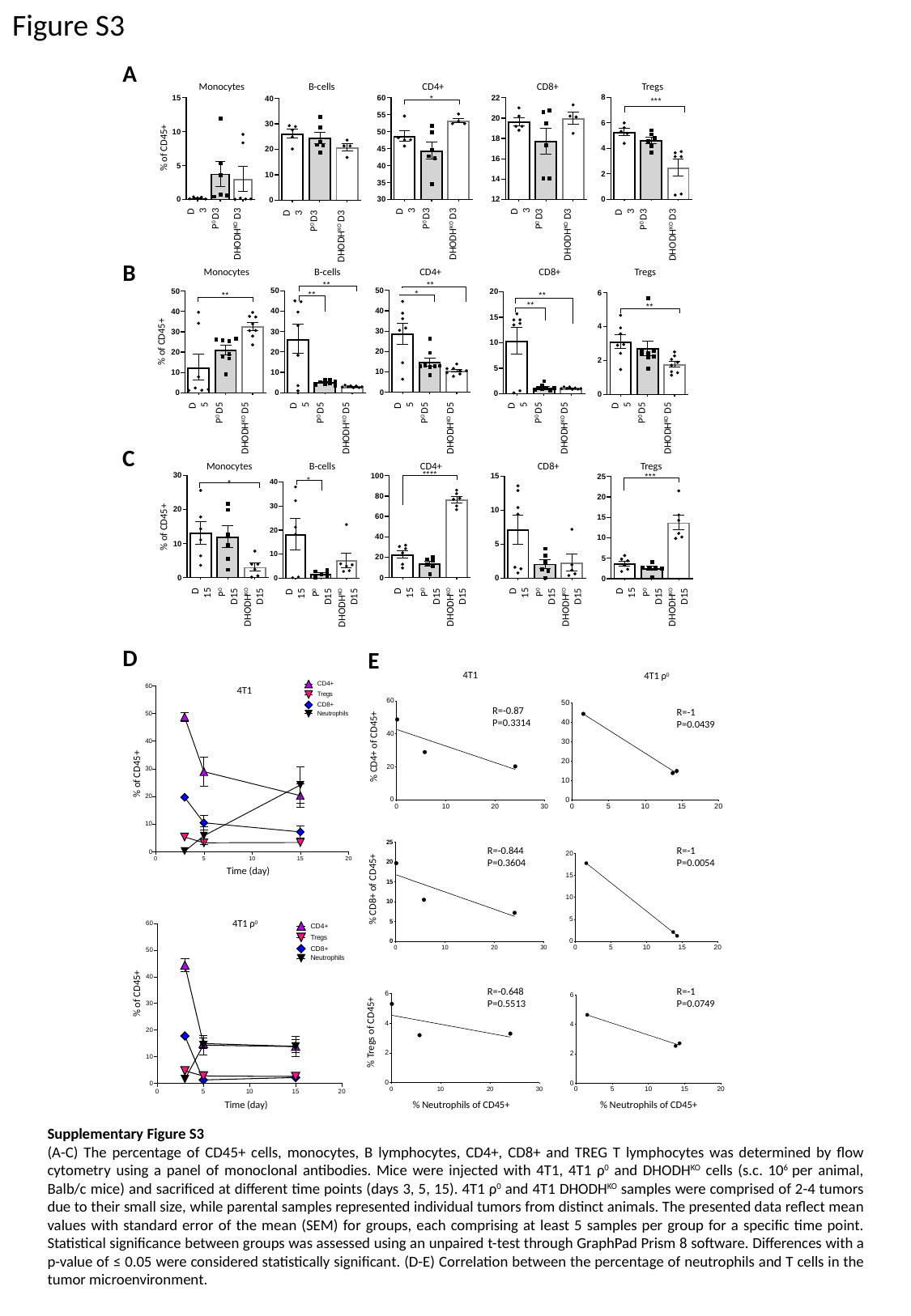

Figure S3
A
Monocytes
B-cells
CD4+
CD8+
Tregs
% of CD45+
D3
Ρ0 D3
DHODHKO D3
D3
Ρ0 D3
DHODHKO D3
D3
Ρ0 D3
DHODHKO D3
D3
Ρ0 D3
DHODHKO D3
D3
Ρ0 D3
DHODHKO D3
B
Monocytes
B-cells
CD4+
CD8+
Tregs
% of CD45+
D5
Ρ0 D5
DHODHKO D5
D5
Ρ0 D5
DHODHKO D5
D5
Ρ0 D5
DHODHKO D5
D5
Ρ0 D5
DHODHKO D5
D5
Ρ0 D5
DHODHKO D5
C
B-cells
CD4+
CD8+
Tregs
Monocytes
% of CD45+
D15
Ρ0 D15
DHODHKO D15
D15
Ρ0 D15
DHODHKO D15
D15
Ρ0 D15
DHODHKO D15
D15
Ρ0 D15
DHODHKO D15
D15
Ρ0 D15
DHODHKO D15
D
4T1
% of CD45+
E
4T1
4T1 ρ0
R=-0.87
P=0.3314
R=-1
P=0.0439
% CD4+ of CD45+
R=-0.844
P=0.3604
R=-1
P=0.0054
Time (day)
4T1 ρ0
% of CD45+
Time (day)
% CD8+ of CD45+
R=-0.648
P=0.5513
R=-1
P=0.0749
% Tregs of CD45+
% Neutrophils of CD45+
% Neutrophils of CD45+
Supplementary Figure S3
(A-C) The percentage of CD45+ cells, monocytes, B lymphocytes, CD4+, CD8+ and TREG T lymphocytes was determined by flow cytometry using a panel of monoclonal antibodies. Mice were injected with 4T1, 4T1 ρ0 and DHODHKO cells (s.c. 106 per animal, Balb/c mice) and sacrificed at different time points (days 3, 5, 15). 4T1 ρ0 and 4T1 DHODHKO samples were comprised of 2-4 tumors due to their small size, while parental samples represented individual tumors from distinct animals. The presented data reflect mean values with standard error of the mean (SEM) for groups, each comprising at least 5 samples per group for a specific time point. Statistical significance between groups was assessed using an unpaired t-test through GraphPad Prism 8 software. Differences with a p-value of ≤ 0.05 were considered statistically significant. (D-E) Correlation between the percentage of neutrophils and T cells in the tumor microenvironment.
