# Supplementary material for: Mitochondrial Transfer Rescues Respiration to Support De Novo Pyrimidine Biosynthesis and Tumor Progression
Source: Cancer Res. 2025 Nov 17;86(4):925–39. doi: 10.1158/0008-5472.CAN-24-0737 (PMC13053058; doi:10.1158/0008-5472.CAN-24-0737)
Supplement: Figure S4 — H&E staining [file can-24-0737_figure_s4_suppsf4.pptx]

## Slide 1
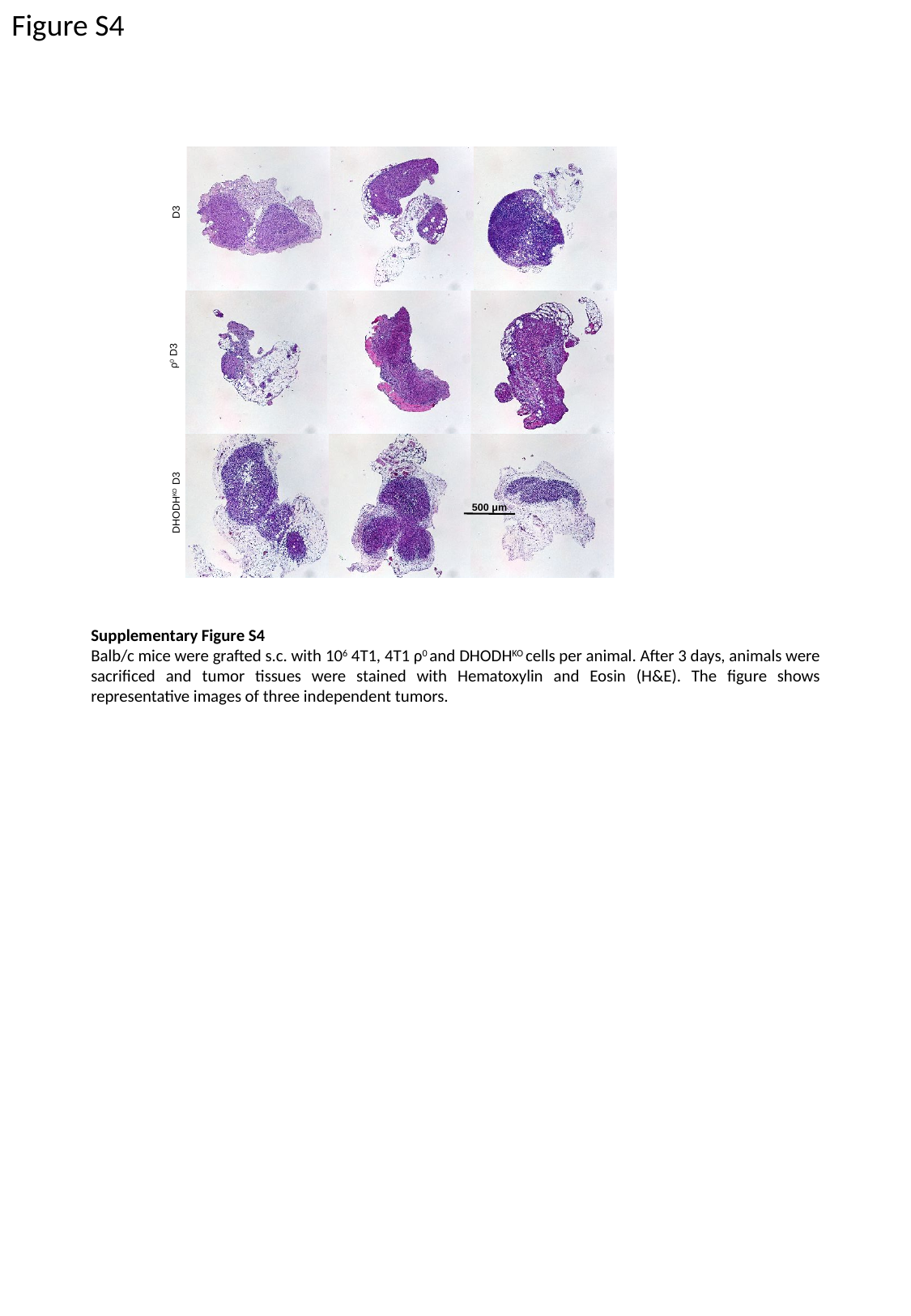

Figure S4
D3
 ρ0 D3
DHODHKO D3
500 μm
Supplementary Figure S4
Balb/c mice were grafted s.c. with 106 4T1, 4T1 ρ0 and DHODHKO cells per animal. After 3 days, animals were sacrificed and tumor tissues were stained with Hematoxylin and Eosin (H&E). The figure shows representative images of three independent tumors.
