# Supplementary material for: Mitochondrial Transfer Rescues Respiration to Support De Novo Pyrimidine Biosynthesis and Tumor Progression
Source: Cancer Res. 2025 Nov 17;86(4):925–39. doi: 10.1158/0008-5472.CAN-24-0737 (PMC13053058; doi:10.1158/0008-5472.CAN-24-0737)
Supplement: Figure S5 — Mitochondrial nucleoids [file can-24-0737_figure_s5_suppsf5.pptx]

## Slide 1
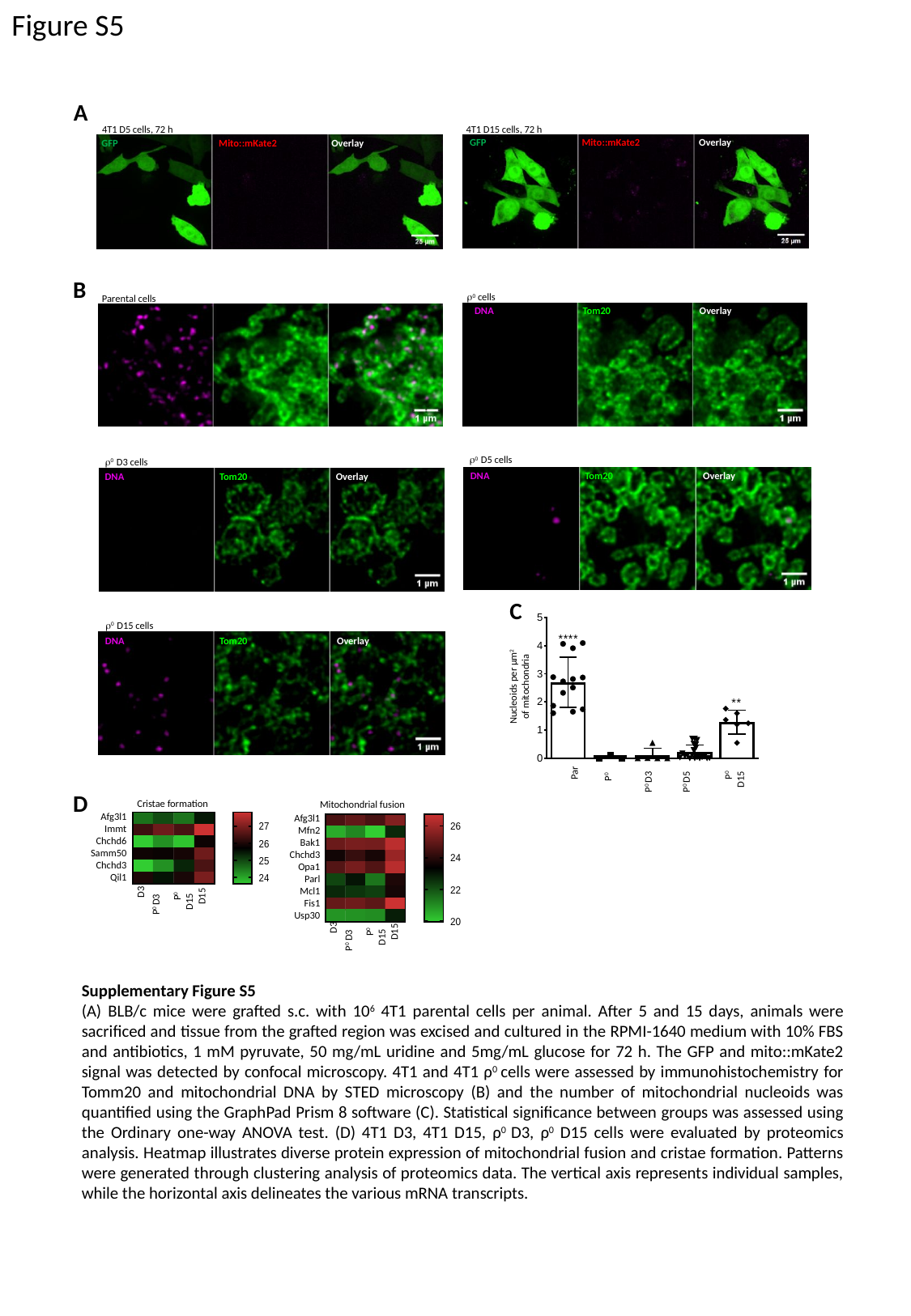

Figure S5
A
4T1 D15 cells, 72 h
4T1 D5 cells, 72 h
GFP
Mito::mKate2
Overlay
GFP
Mito::mKate2
Overlay
B
r0 cells
Parental cells
Tom20
Overlay
DNA
Tom20
Overlay
DNA
r0 D5 cells
r0 D3 cells
Tom20
Overlay
DNA
Tom20
Overlay
DNA
C
Nucleoids per µm2 of mitochondria
Ρ0
Ρ0 D3
Ρ0 D5
Ρ0 D15
Par
r0 D15 cells
DNA
Tom20
Overlay
D
Cristae formation
Afg3l1
Immt
Chchd6
Samm50
Chchd3
Qil1
D3
Ρ0 D15
Ρ0 D3
D15
Mitochondrial fusion
Afg3l1
Mfn2
Bak1
Chchd3
Opa1
Parl
Mcl1
Fis1
Usp30
D3
Ρ0 D15
Ρ0 D3
D15
Supplementary Figure S5
(A) BLB/c mice were grafted s.c. with 106 4T1 parental cells per animal. After 5 and 15 days, animals were sacrificed and tissue from the grafted region was excised and cultured in the RPMI-1640 medium with 10% FBS and antibiotics, 1 mM pyruvate, 50 mg/mL uridine and 5mg/mL glucose for 72 h. The GFP and mito::mKate2 signal was detected by confocal microscopy. 4T1 and 4T1 ρ0 cells were assessed by immunohistochemistry for Tomm20 and mitochondrial DNA by STED microscopy (B) and the number of mitochondrial nucleoids was quantified using the GraphPad Prism 8 software (C). Statistical significance between groups was assessed using the Ordinary one-way ANOVA test. (D) 4T1 D3, 4T1 D15, ρ0 D3, ρ0 D15 cells were evaluated by proteomics analysis. Heatmap illustrates diverse protein expression of mitochondrial fusion and cristae formation. Patterns were generated through clustering analysis of proteomics data. The vertical axis represents individual samples, while the horizontal axis delineates the various mRNA transcripts.
