# Supplementary material for: Mitochondrial Transfer Rescues Respiration to Support De Novo Pyrimidine Biosynthesis and Tumor Progression
Source: Cancer Res. 2025 Nov 17;86(4):925–39. doi: 10.1158/0008-5472.CAN-24-0737 (PMC13053058; doi:10.1158/0008-5472.CAN-24-0737)
Supplement: Table S1 — Flow cytometry antibodies [file can-24-0737_table_s1_suppst1.docx]

**Table S1.** Flow cytometry antibodies

| **Antigen** | **Fluorochrome** |  | **Clone ID** | **Catalog #** | **Manufacturer** | **RRID** | **dilution** |
| --- | --- | --- | --- | --- | --- | --- | --- |
| Bst2 | PE-eF610 |  | eBio927 | 61317282 | Thermo Fisher Scientific | AB_2574604 | 1:800 |
| CD4 | BV650 |  | RM4-5 | 563747 | BD Biosciences | AB_2716859 | 1:1500 |
| CD5 | BV421 |  | 53-7.3 | 562739 | BD Biosciences | AB_2737758 | 1:300 |
| CD8a | BUV395 |  | 53-6.7 | 563786 | BD Biosciences | AB_2732919 | 1:400 |
| CD11b | PerCP-Cy5.5 |  | M1/70 | 101228 | BioLegend | AB_893233 | 1:800 |
| CD11c | BUV737 |  | HL3 | 612797 | BD Biosciences | AB_2870124 | 1:200 |
| CD19 | BV510 |  | 1D3 | 562956 | BD Biosciences | AB_2737915 | 1:300 |
| CD24 | BUV563 |  | 30-F1 | 752795 | BD Biosciences | AB_2917775 | 1:300 |
| CD25 | PE-Cy7 |  | PC61 | 552880 | BD Biosciences | AB_394509 | 1:200 |
| CD31 | BUV805 |  | MEC 13.2 | 741939 | BD Biosciences | AB_2871251 | 1:400 |
| CD45 | FITC |  | 30 F11 | 103108 | BioLegend | AB_312973 | 1:800 |
| CD49f | APC-Cy7 |  | [GoH3](https://www.biolegend.com/en-us/search-results?Clone=GoH3) | 313627 | BioLegend | AB_2616783 | 1:300 |
| CD90 | BUV615 |  | 30-H12 | 751613 | BD Biosciences | AB_2875607 | 1:1000 |
| CD206* | BV605 |  | C068C2 | 320014 | BioLegend | AB_439750 | 1:100 |
| DX5 | PE |  | DX5 | 108907 | BioLegend | AB_313414 | 1:400 |
| EpCAM | BV480 |  | G8.8 | 746367 | BD Biosciences | AB_2743684 | 1:600 |
| F4/80 | PE-Dazzle594 |  | BM8 | 123146 | BioLegend | AB_2564133 | 1:300 |
| Fc block |  |  | 2.4G2 | 553142 | BD Biosciences | AB_394656 | 1:200 |
| FoxP3* | AF647 |  | 150D | 320014 | BioLegend | AB_439750 | 1:200 |
| Ly6C | AF700 |  | AL-21 | 561237 | BD Biosciences | AB_10612017 | 1:300 |
| Ly6G | BV785 |  | 1A8 | 127645 | BioLegend | AB_2566317 | 1:400 |
| MHCII | BB630 |  | M5/114.15.2 | 624294 | BD Biosciences | custom conjugate | 1:800 |
| Sca1 | BV711 |  | D7 | 563992 | BD Biosciences | AB_2738529 | 1:1000 |
